# Bio Meets Nano: Protein Exchange in Saline Biocoronae on Magnetic Nanoparticles

**DOI:** 10.3390/ijms26188995

**Published:** 2025-09-16

**Authors:** Paula Fraga-García, Sandra Haßelt, Carlos Eduardo Díaz-Cano, Lucía Abarca-Cabrera, Yasmin Kaveh-Baghbaderani, Sebastian P. Schwaminger, Massimo Kube, Hendrik Dietz

**Affiliations:** 1 Chair of Bioseparation Engineering, Department of Energy & Process Engineering, School of Engineering & Design, Technical University of Munich, 85748 Garching, Germany; sandra.hasselt@gmail.com (S.H.); cadiazcano@gmail.com (C.E.D.-C.); l.abarca@tum.de (L.A.-C.); y.kaveh@tum.de (Y.K.-B.); 2 NanoLab, Division of Medicinal Chemistry, Otto-Loewi Research Center, Medical University of Graz, 8010 Graz, Austria; sebastian.schwaminger@medunigraz.at; 3 BioTechMed-Graz, 8010 Graz, Austria; 4 Lehrstuhl für Biomolekulare Nanotechnologie, Department of Biosciences, School of Natural Sciences, Technical University of Munich, 85748 Garching, Germany; massimo.kube@tum.de (M.K.); dietz@tum.de (H.D.)

**Keywords:** bio-nano interface, bio-nano interactions, biocorona, biomolecule adsorption, iron oxide nanoparticles, magnetic separation, bioseparation, bionanotechnology, protein recovery, protein separation

## Abstract

When iron oxide nanoparticles are incubated together with a biological broth, the biomolecules compete for the binding sites at the solid–liquid interface. At the same time, the biomass rearranges in suspension, building agglomerated structures. Despite general knowledge of the forces involved in bio–nano interactions, gaps remain in the understanding of how biomolecules organize themselves in solution and onto surfaces. This work examines biomolecule adsorption onto metal oxide surfaces with the goal of strengthening this understanding, essential in industrial and natural processes. We demonstrate nearly complete separation of proteins from a biotechnological suspension for non-oxidized and highly oxidized magnetic nanoparticles. Varying the nanoparticle-to-biomass ratio, we find, can lead to different separation patterns, i.e., that selectivity using bare, low-cost materials is possible. Furthermore, we explore how preliminary “passivation” with a biological corona only partially reduces the ability to separate total protein mass from a new suspension in subsequent incubation steps. The study underscores the crucial role of concentration gradients with regard to targets and binding sites as the primary determinant of separation capacity and of biomolecule behavior in solution, highlighting the potential for using bio–nano coronae as biomolecule carriers across diverse fields, including environmental, biomedical, pharmaceutical and nutritional applications.

## 1. Introduction

Interfacial phenomena continue to be as challenging as when Wolfgang Pauli made his famous declaration, “God made the bulk; the surface was invented by the devil” [1]. Despite copious research on all types of interfaces over the decades in areas such as catalysis, biotechnology, separation processing, biogeochemistry and drug delivery, just to name a few, knowledge of the driving mechanisms behind interfacial processes continues to be incomplete and is still often mainly based on empirical work [2,3]. A myriad of life science applications, e.g., biosensing, targeted therapies and tissue engineering as examples from the biomedicinal field, depend on interfacial phenomena like protein adsorption, cell adhesion and ion exchange at the interface between biological fluids or tissues and inorganic substrates [4]. Magnetic nanoparticles (MNPs) comprise one specific type of solid surface and are particularly attractive due to the combination of their superparamagnetism with the common high surface-to-volume ratio of all nanomaterials. MNPs have experienced a continuous boom in the number of applications with special emphasis on biomedicine [5,6,7], where potential toxicity issues still need to be solved [8], as well as wastewater treatment and nanoelectronics [9]. However, the range of possibilities is far broader. Bioseparations represent an additional important application field with enormous prospects for advancement, from downstream processing to the nutritional and pharmaceutical industries [10].

When the solid surface of a nanoparticle meets a liquid biological environment, an exciting phenomenon takes place: a protein corona grows on the nanoparticles [11,12,13,14,15]. Protein coronae have been a hot topic for almost two decades [16,17], mainly in biomedicine and drug delivery studies [18,19], but with increasing attention in environmental [20,21], agricultural [22] and biotechnological research [23,24]. Wheeler et al. define the protein corona as a “layer of proteins that spontaneously adsorbs to the surface of engineered nanomaterials” [20], and Mohammadi et al. define it as “a spontaneous opsonization and absorption of active biomolecules, i.e., lipids, metabolites, sugars, and proteins” of NPs entering the body [25]. The literature on the protein corona is squarely focused on the animal physiological protein corona: blood serum or plasma are the main environments chosen [26]. In contrast, the protein corona from biotechnological lysates remains little studied [23]. Even if studies on bio–nano interactions are normal in biotechnology and related fields, they primarily analyze the behavior of one model molecule, and rarely more than two target molecules in competition, sometimes from a mixture, but there is scarcely any general knowledge of the behavior of complex mixtures interacting with magnetic nanoparticles [23].

The persistent lack of information on the “biotechnological” coronae compared to the published work on coronae from animal environments (mainly blood) might be due to the complexity of the problem: the number of organisms, as well as the number of possible environments, is far higher, and the same can be said about the number of molecules and ions present, as well as their diversity compared to the ones only in blood or serum plasma. The biological environment is generally well known only for intensively studied systems such as *E. coli* cell debris. The complexity of biotechnological systems unavoidably leads to a complexity in the analytics, which are the essential tool for obtaining data and therefore for increasing understanding of the mechanisms that control corona formation and transformation [27]. Nevertheless, industrial biomolecule production using microorganisms is becoming increasingly important. In order to separate the target product as selectively and purely as possible from the cellular soup, separation with magnetic nanoparticles is an effective method because of the advantageous usage of magnetic forces that simplify processing and make possible an increase in processing volumes and a decrease in processing time [10].

The recent work by Gurtu et al. [28] is a recent example of a study focusing on the biological corona outside physiological environments; the authors discuss the effect of different surfaces together with the influence of different kinds of molecules, but do not examine the impact of concentration gradients on the protein footprint. In our research, we have learned that the quantity of adsorbed proteins in protein–nanoparticle incubation experiments depends strongly on the concentration ratio of all species present [23]. In a previous work, we discovered that microalgae cells could be almost completely harvested from a liquid phase when we deployed enough magnetic nanoparticles [29]. In a recent publication, we demonstrate that the same can be achieved with proteins and lipids, and that even very soluble lipidic and proteinic species can be separated with a moderate quantity of magnetic nanoparticles [23]. The present work is closely related to one recently published by Abarca-Cabrera et al. [23], which focused on the bigger corona picture. In the current manuscript, we clarify additional issues related to the nanoparticle-to-protein mass ratios and to sequential incubation steps (with an “intermediate” corona), but concentrate only on proteins, which are easier to quantify. We focus here again on the protein corona of a sea water microalgae species. Sea water microalgae are particularly important, among other uses, because their growth and processing does not require precious potable water [30,31]. Microalgae cultivation is the focus of extensive research, showing encouraging improvements in production efficiency [32,33] and in large-scale disruption [34]. Notably, open thin-layer cascade photobioreactors, considered more cost-effective for large-scale operations, have achieved cell concentrations reaching up to 50 g per liter [35]. In the last few years, their value as a lipid source for biofuels, and also in nutritional, biomedicinal, pharmaceutical and cosmetic areas, has been recognized [36,37,38]. The current study is based on *Microchloropsis salina* cells (former *Nannochloropsis salina*), an interesting source of biofuels and high value products (e.g., antioxidants and polyunsaturated fatty acids) owing to their high biomass productivity and lipid content, further enhanced in recent years through metabolic and/or genetic engineering [39,40]. A remaining bottleneck is the lack of efficient procedures for large scale separating, concentrating and dewatering microalgae [41,42,43]. Here, magnetic separation provides an opportunity to enhance processing and reduce costs [42], and offers interesting prospects for microalgae fractionation.

This work is a small step forward in elucidating how complex biological (and, more particularly, biotechnological) mixtures interact with nanoparticulate systems, and how a biomass distributes itself between the solid/liquid interphase and the solution. Above, we introduced magnetic separation using nanoparticles, the corona topic and microalgae as a source of biomolecules. In the following sections, we seek an answer to the question of whether the corona building process leads to some kind of selectivity, and whether, after equilibration of the corona “intermediate” with the environment, the dynamics of the corona still lead to a high degree of exchange with a new protein source in the media. The data show how different concentration gradients influence the mass of protein separated from a microalgae cell lysate, as well as their influence on single protein adsorption. Furthermore, the behavior of a one-protein corona is compared to a complex biocorona. This study aims to deepen the general understanding of protein corona mechanisms and pave the way for biomolecule fractionation in natural environments using magnetic nanocarriers.

## 2. Results and Discussion

As described in the introduction, there is a lack of biomolecule adsorption data for solids in biotechnological mixtures, model or real. To address this gap, we used *Microchloropsis salina* (*M. salina*) microalgal lysate as a biological source in the experiments here.

### 2.1. Quantitative Protein Separation

We work intensively with magnetic nanoparticles, which are superparamagnetic. We produce magnetite using a previously established synthesis [44,45], which is based in the Massart synthesis method [46,47]. However, the particles oxidize with time and depending on their history, i.e., depending on environmental conditions such as pressure, temperature and the media pH. Comparing magnetite (Fe_3_O_4_) and its oxidized form, maghemite (γ-Fe_2_O_3_), we wanted to discover if the oxidation of the surface impacts the separated protein mass while maintaining a very similar particle size. The oxidation question was important to us because the particles become older with time, and they often then change their color from black to brownish. With an eye on processes based on these types of magnetic iron oxides, we wanted to clarify if this oxidation might impact their separation capacity. Both magnetite and maghemite are low-cost materials that can be easily synthesized sustainably [45,48], two important advantages for industrial-scale applications. Furthermore, their most exciting and special property is probably their superparamagnetism. Details on the particles’ synthesis and characterization methods are described in the Supplementary Materials. Size, infrared, magnetization and diffraction data of magnetite are presented in Figure S1. Before the incubation experiments, the particles were stored in artificial seawater (ASW, a highly saline media; see details on composition in the Supplementary Materials). The main characteristics of the particles relevant for this work are the crystal size of the individual crystalline units and sufficient magnetization to be easily separated using a hand magnet. Key data are as follows:

- Magnetite nanoparticles (BIONs): size, 8.9 nm diameter measured via transmission electron microscopy (TEM) and 9.2 nm via X-ray diffraction (XRD); saturation magnetization, 70 emu g^−1^. See Figure S1 for characterization data.
- Maghemite nanoparticles: size 9 and 7 nm from TEM and XRD data, respectively; saturation magnetization, 66 emu g^−1^ [48].

**Figure 1:**
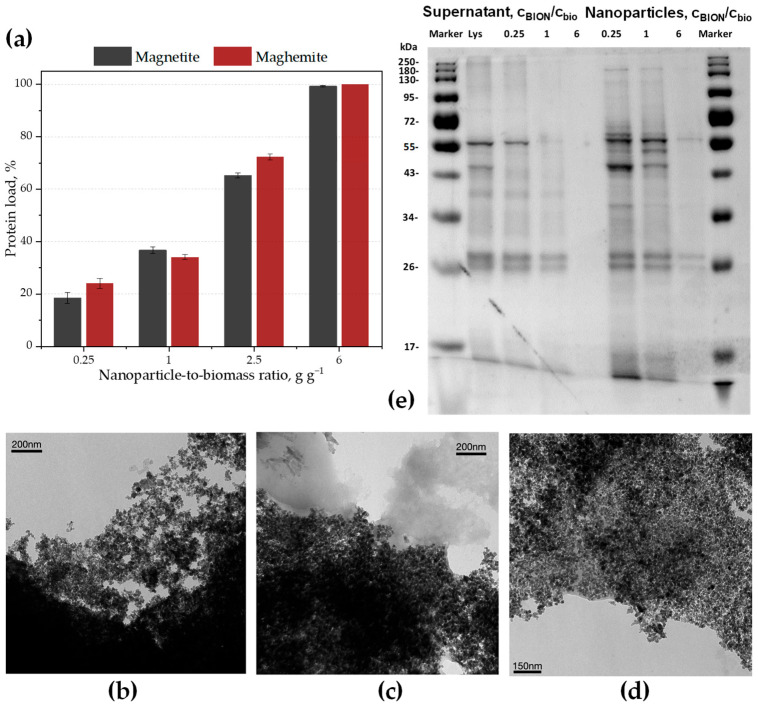
(**a**) Percentage of total protein adsorption from microalgal lysate onto iron oxide nanoparticles for different nanoparticle-to-biomass ratios; (**b**) BIONs in artificial seawater (ASW); (**c**,**d**) BIONs incubated with microalgal lysate at nanoparticle-to-biomass ratios of 0.25:1 g g^−1^ and 6:1 g g^−1^, respectively; (**e**) SDS-PAGE of protein bands in the lysate (Lys), in the supernatant, and adsorbed onto the nanoparticles, obtained after incubation of microalgal lysates and BIONs at mass ratios of 0.25:1, 1:1 and 6:1 BION-to-biomass.

We sought to determine how the concentration of protein in solution evolves with increasing nanoparticle-to-biomass mass ratios, and if the separated protein mass depends on the oxidation of the nanoparticle surface. Therefore, we carried out experiments incubating the two kinds of nanoparticles described above with an *M. salina* microalgal lysate at different mass ratios of nanoparticles-to-biomass. In Figure 1a, we show data on the separated total protein mass compared to the initial mass in the biological suspension before magnetic separation.

Figure 1a demonstrates that when the nanoparticle mass increases, the fraction of separated proteins also increases, and a mass of nanoparticles can be found which enables magnetic separation of almost all proteins attached to the solid phase. This confirms recently published data where we have demonstrated the almost complete separation of proteins and lipids by means of magnetic nanoparticles [23], and expands its validity from magnetite to maghemite nanoparticles. Figure 1b shows the BIONs (magnetite) in ASW media, and in Figure 1c,d the biocorona adsorbed onto the BIONs, after washing the particles twice with deionized water, can be observed. Images of the lysate can be found in the Supplementary Materials Figure S2. Figure 1c,d correspond to the two extreme cases: Figure 1c shows the corona for a very low BION-to-biomass ratio (0.25:1 g_BION_ g_biomass_^−1^), whereas Figure 1d shows it for a very high ratio (6:1 g_BION_ g_biomass_^−1^). At the low ratio, there are a greater number of proteins than the available surface, while at the high ratio more surface area is available than the number of proteins in the system. The general picture from TEM images is not that different among the two extreme situations. No substantial differences are observable, although the quantity of adsorbed material per iron oxide unit mass differs substantially. In both cases, a cloud of biomolecules surrounding the nanoparticles can be observed in several areas of the images, and at the same time a pale coating surrounding the dark nanoparticle spots seems to correspond to the thin layer of biological material distributed onto the solid surface.

We expected that oxidation of the nanoparticles would not significantly affect the total mass of proteins separated by magnetic fishing, which is confirmed by the data in Figure 1a. Even if corona formation is known to take place differently on very different surfaces [49,50], the differences for similar oxides might not be that significant, and the corona composition probably depends chiefly on the biological environment. Our data show that the mass-to-mass evolution of the corona develops in a similar form no matter how oxidized the material is. We primarily wanted to determine with these experiments whether the oxidized nanoparticles, as a “more passivated” surface with potentially less surface activity, would be less capable of separating large protein masses. This does not seem to be the case, possibly because the direct interface, the first solid interfacial layers, is not that different in its activity with regard to the two materials we compare here. De Castro et al. also observed a reproducible corona size and growth on different synthetic polymer nanoparticles [51].

Figure 1a, interestingly, also shows the large quantity of proteins that can be separated. In this case, taking into account the protein percentage of roughly 25% of the total biomass, 6 g magnetite is necessary to separate about 0.25 g protein from a lysate. We found published values for clay minerals as high as 0.5 g protein per g montmorillonite [52,53], which demonstrate the incredibly high capability of mineral materials to incorporate large quantities of proteins. The published data are in a similar order of magnitude to the protein mass separated by our nanoparticles. Note here that a common montmorillonite clay bulk density is in the range 2–3 g cm^−3^ [54], while the value for magnetite is 5.2 g cm^−3^ [55], and for maghemite 4.9 g cm^−3^ [55]. Comparing this data suggests that the particular surface chemistry is not the most decisive factor. The fact that inorganic crystals generally have high surface energies leads to the assumption that the total masses of accumulated organic mass per solid surface unit for similar biological environments (similar type-of-molecules distribution and ionic strength conditions) should not differ strongly, or should at least be in a similar dimension. Factors such as the charge and roughness of the surface, the homogeneity of the topography, morphological issues and purity of the solid (often crystalline) phases are certainly further important parameters influencing the total accumulated protein mass. Moreover, Weiss [53] describes the accumulation of proteins along very long time frames (in connection with crude oil reservoirs). This accumulation appears to be a far more complex process, much more influenced by, e.g., pressure and time than the one we have studied here. Furthermore, we hypothesize that the growth of the coronae in our experiments might be facilitated by the high ionic strength of the saline media from the algae lysate. It is established knowledge in the colloids community that high ionic strength leads to the shielding of double layer forces, leading to a larger van der Waals share in the total interaction potential, and therefore to agglomeration and coagulation, according to the DLVO-Theory (DLVO for Derjaguin–Landau–Verwey–Overbeek) [56,57].

Additionally, the increase in ionic strength leads in many cases to a reduction in molecule solubility. We have recently shown for amino acids with very close isoelectric points that lower solubility was connected with increased partitioning of the target molecule for the solid support, and therefore yielded a higher target capacity for the same nanoparticle-to-biomolecule mass ratio for the conditions in the experiment and for low analyte concentrations in solution [58].

### 2.2. Potential to Find Predominating Interactions

One essential objective which drives our experimental work is to determine whether particular proteins have a specificity for the solid surface, which in biotechnology is often called affinity, and whether there are indications that allow us expect selectivity using bare, non-functionalized, low-cost, high surface-to-volume iron oxides. A related set of questions is whether the corona itself “bares” some kind of specificity: are some proteins less prone to form a hard or a soft corona? Are there some proteins which do not form a corona or become part of a corona, for instance, because they are highly soluble and are more likely to remain in water? A first step in the search for answers is to simply compare the protein bands from molecules remaining in solution to the ones forming a corona after an incubation experiment. Accordingly, we analyzed the corona and the supernatant proteins in the experiments with the BIONs from Figure 1a using SDS-PAGE (see Figure 1e) to learn if the ratio between the number of proteins and the accessible surface, i.e., the number of available binding sites, somehow changed the profile of the proteins adsorbed onto the solids. In these experiments, this ratio is defined by the proportion of nanoparticles to biomass. From Figure 1a, we could confirm that the mass-related behavior does not depend on the degree of oxidation. Therefore, we decided to focus our additional work only on the less-oxidized particles, because we obtain them directly after synthesis without further modification. These are the particles we call BIONs [29]. Here, we want to point out that the abbreviation BION (bare iron oxide nanoparticle) could work for pure magnetite, pure maghemite and all “intermediate” states, even if we use it only for magnetite particles synthesized following our recipe [44,45]. Magnetite has a small advantage over maghemite for bioseparation processing due to its slightly higher saturation magnetization [48].

Comparing the bands of the proteins remaining in a solution after incubation with those adsorbed onto the surface, and, moreover, comparing the changes in their intensities depending on the nanoparticle concentrations, we expected to establish if there could be room for specific differences within proteins related to their partitioning between the corona and the solution. Indeed, some bands in Figure 1e show clear differences in intensity between the lysate lane, the supernatant lanes and the nanoparticle lanes. These differences indicate a preference for adsorption of specific proteins onto the nanoparticles. Specific bands in this regard are highlighted in the Supplementary Materials Figure S3. Comparing the protein bands on the BIONs to the bands from the supernatant, particularly in the 35 to 55 kDa region and for low particle-to-biomass ratios, clear effects can be observed. Some bands not visible in the lysate lane appear on the particle surface; others are pale in the lysate and more intense on the particles; and still others, in turn, are intense from the supernatants but pale on the nanoparticles. The disappearance of a band from the lysate gel that only reappears on the particles’ gel and not on the supernatant gel, for instance, indicates that there may be a selectivity in the interaction between the corona or the surface of the nanoparticles and certain types of proteins. This is a first indicator that some proteins might have a preference for one type of surface corona over others, either because of their charge, their structure-building ability, their hydrophobicity or their solubility. For the results in Figure 1e, we had incubated BIONs and biomass for 1 h. We assume that the concentration profile changes over time, but we have not yet analyzed this, even if the topic of the process kinetics is very important. Based on our results, we hypothesize that solubility is an essential player and might even be a decisive factor in the selectivity question, as we have previously argued [23,59]. Recently, we demonstrated the impact of solubility on partitioning and adsorption capacity on the particles for several amino acids [58]. We expect that the more soluble the proteins are, the lower their tendency to leave the solution and adsorb onto the surface is, i.e., the lower their partitioning towards the solid phase. Again, the concentration profile of the target analyte and the BIONs will define the ratio of adsorbed analyte molecules. If the number of binding places is high enough (high BION concentration compared to the biomolecule number), all proteins, no matter whether they are more or less soluble, will adsorb onto the surface. If the number of binding sites is lower (lower BION concentration in comparison with the situation described above), some biomolecules will prefer to remain in solution. From the band differences between the SDS gels on the particles and in the supernatants in Figure 1e, we observe that differences exist between the proteins’ partitioning, depending on the number of binding sites available. Unfortunately, our analytical capabilities do not enable us to analyze the proteins of the individual bands at the moment. Many microalgae proteins have not even been identified, and it is very difficult to track down the literature on particular proteins from this kind of microorganism. Nevertheless, we hypothesize that RuBisCo and proteins from the light-harvesting complexes (LHC) are recognizable in the gels, based on the previous literature [23]. RuBisCo’s light and heavy chains should be found at approx. 15 and 55 kDa, respectively [60,61], and the LHC bands between 20 and 45 kDa [23,62,63,64].

At the highest nanoparticle-to-biomass ratio (e.g., 6:1 in Figure 1e), all available protein is expected to bind to the particles, i.e., no selective binding is possible under these conditions but the separation of all the protein mass. Therefore, we focused on analyzing the lanes with lower nanoparticle-to-biomass ratios (0.25:1 and 1:1) and normalized the results to the amount of particles used. Accordingly, we had the same nanoparticle masses in the three nanoparticle lanes. At higher mass-to-mass ratios, less protein is bound per particle, leading to the absence of visible protein bands in the lanes corresponding to the highest ratio, as seen in Figure 1e.

### 2.3. Protein Exchange Between the Corona and the Environment

We are interested in understanding the exchange dynamics of the soft corona, and we study this employing the model molecule Green Fluorescent Protein (GFP) which we have used in previous studies [65,66], as it is simple to quantify due to its fluorescence. We seek to determine the influence of the addition sequence of the components on the corona composition and in dependence to the nanoparticle-to-protein ratio. When a biological corona already covers the surface, is it still possible to adsorb large protein quantities? How extensive is the protein exchange of an already formed biocorona with the surroundings? To answer these questions, we compare two situations here: (1) the initial incubation of GFP with the BIONs (GFP corona), followed by the subsequent incubation of the corona “intermediate” in a microalgae lysate; and (2) the incubation of the lysate with the BIONs (lysate corona), followed by the addition of a GFP solution to the corona “intermediate”. These experiments should enable us to understand how dynamic the corona is, and if, after its equilibration with the surroundings, a new environment leads to a significant exchange of molecules between the existing corona and the new solution. Additionally, we wanted to determine whether, after saturation of the surface in the form of a corona, the addition of new proteins leads to further exchange. The primary question guiding these experiments is as follows: does the formed corona intake a large number of additional proteins if new proteins are subsequently added to the media? The corona “intermediate” was washed twice with ASW media prior to the next incubation.

**Figure 2:**
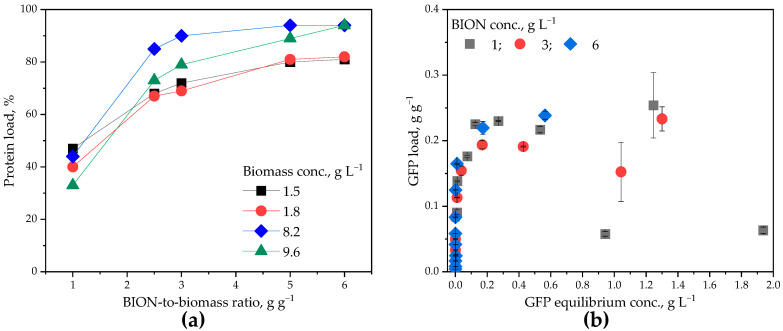
(**a**) Percentage of protein mass adsorbed. Two different batches of microalgae lysate (1.5 and 1.8 g L^−1^) and their concentrated fractions (8.2 and 9.6 g L^−1^, respectively) as a function of the BION-to-biomass ratio in g g^−1^. The amount of adsorbed protein increases with increasing BION-to-biomass ratio until entering a saturation region close to 6 g g^−1^. (**b**) GFP adsorption isotherms for different BION-to-protein ratios. BIONs concentration of 1, 3 and 6 g L^−1^. GFP load in g_GFP_ g_BION_^−1^.

As preliminary experiments to the sequential runs, we repeatedly quantified the quantity of proteins adsorbed related to the mass of BIONs with two new algae charges and algae concentrations to gain an insight into the differences among batches. The data in Figure 2a corroborate the correlation between separated proteins and the BION mass, presented in the previous section, for new microalgae batches, and demonstrate that this correlation does not significantly depend on the total algae concentration present in the suspension, at least for the selected range. Even for biomass concentrations close to 10 g L^−1^ a similar quantity of proteins can be separated if the ratio of BIONs to biomass is kept constant. The two highest biomass values originate from the two lowest ones after concentration of the biomass through centrifugation. The data in Figure 2a demonstrate the robustness of the separated protein mass for different algae batches (different ages, slightly different concentrations of proteins), an important characteristic from an industrial processing point of view due to the variability in biotechnological cultivations. The data confirm again the data contained in the previous section and in our recent publication [23]. For processing goals, the separated protein mass must be independent of the total biomass concentration in suspension for the range of concentrations studied. The higher concentrations are close to common values in high yield microalgae cultivation, even if a few reports on higher biomass values can also be found [35]. Figure 2a shows a slight increase in separated protein mass for lower pH, as is the case for the two higher biomass concentrations. While the pH of the original algae batches was approx. 7.8–8.4, the concentrated ones had a lower pH of approx. 6.6. We had observed the effect of higher loading for lower pH in previously published studies [23,52], a tendency that has been generally reported for different mineral materials [52]. Nevertheless, the slightly higher loadings for higher total biomass concentrations might also be due to agglomeration effects or to differences in the initial protein concentrations.

The next step was to characterize the adsorption of GFP onto the BIONs, something we had performed in the past, but not in a highly saline media such as ASW and only for a defined BION concentration. As can be seen in Figure 2b, the protein saturation capacity was in the expected range of ~200–400 mg g^−1^. We have observed this range for different proteins on our BIONs during the last few years: maximal loadings of 200–400 mg g^−1^ for “average size” proteins [59,65,67,68], and slightly higher for antibodies [69,70], which are much larger proteins. Our capacity values are higher than those of Hung et al. for bare iron oxide nanoparticles with a specific surface area similar to our BIONs [71]. However, Hung et al. carried out their experiments in another environment, in PBS buffer. We have not represented the values attached in the framework of any adsorption model because we do not consider applying a model particularly helpful here. There is a very steep increase in the capacity for very low protein concentrations (dilution limit) in solution and enough binding sites in the suspension that would lead to undefined slopes. Moreover, upon saturation, the error range increases and the system does not seem to be in an unambiguous equilibrium. As we have already pointed out in the past [69], for low protein concentrations in solution and the right working conditions, most of the proteins are adsorbed onto the nanoparticles. The number of proteins that remain in solution is thus below the analytical sensitivity, and therefore the values in the abscissa tend to zero. Accordingly, presenting the isotherms of adsorbed molecules as a function of the molecules in solution in equilibria makes limited sense, which is why we consciously chose not to apply the isotherm data to a specific saturation model.

In a very recent publication, we calculated the loading of a monolayer of bovine serum albumin (BSA) on BIONs [59] and observed that the range of 200–400 mg_protein_ g_BION_^−1^ corresponds to a more or less fully packed monolayer for mean protein sizes. GFP has a lower molecular weight than BSA (28 kDa) [72]. The divergence of the capacity values for high protein concentrations (>1 g L^−1^ in the supernatant equilibrium concentration) with a clear reduction in capacity is difficult to explain at the moment. The simplest explanation would be an analytical error due to the small volumes and the high degree of dilution necessary at high concentrations in solution in order to measure the sample within the analytical calibration range. Another simple explanation would be the increasing nanoparticle and protein agglomeration effects for the higher concentrations leading to decreased accuracy in the protein quantification. Agglomeration plays an important role in these kinds of processes and leads, in some conditions, to a reduction in the separation capacity of the nanoparticles. Figure S3 validates, and visualizes with SDS gels, the decreasing GFP concentration in solution for the isotherm points. In the samples where the protein concentration was too low to be measured in the supernatants through BCA quantifications, no bands were observed in the gels either.

**Figure 3:**
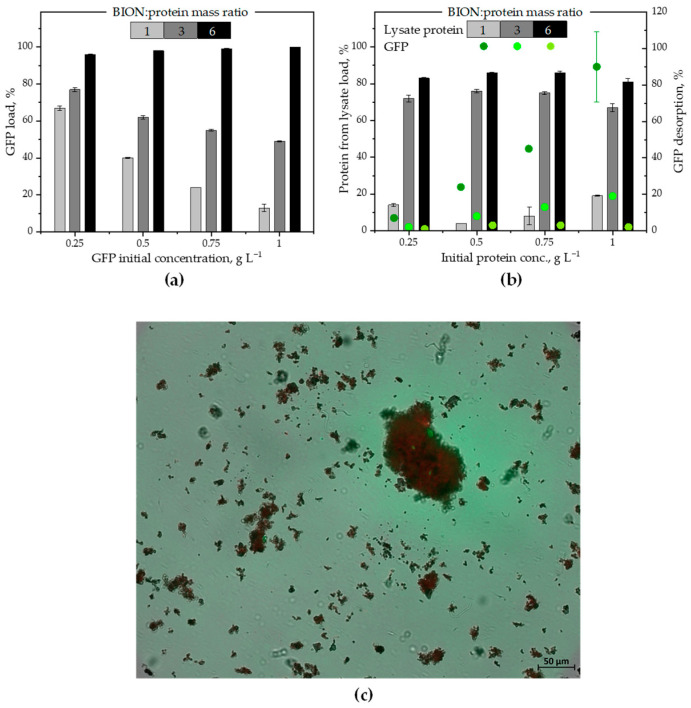
(**a**) Percentage of GFP adsorption onto BIONs for different initial GFP concentrations in solution and for three different BION-to-GFP mass ratios. (**b**) Percentage of total protein adsorption after incubation of the BIONs-GFP corona “intermediate” with microalgal lysate (left ordinate), and percentage of desorbed GFP from the BIONs-GFP corona (right ordinate; the green color dots correspond to the different mass ratios) for the three BION-to-GFP mass ratios from (**a**). The adsorbed protein percentage refers to the initial protein mass in the system. (**c**) Microscopy image of the final solid sample for the BION-to-GFP mass ratio 3:1, first incubated with GFP (0.25 g_protein_ L^−1^) and then with microalgae lysate (0.25 g_protein_ L^−1^) at 200× magnification. Overlapping image combining brightfield (to see the particle agglomerates) with 508 nm fluorescence (to show GFP, in green) and with 685 nm fluorescence (to see chlorophyll, in red). Scale bar 50 µm.

The next step was commencing with the sequential incubation steps. We chose three nanoparticle-to-protein mass ratios to observe the impact of the availability of surface on the separated protein mass. Note that here we refer to the total protein concentration in the lysate and not to the total biomass concentration. We started incubating the BIONs with GFP in ASW, and, after building a GFP corona in equilibrium with the surrounding media and twice washing it with ASW, the BIONs-GFP were incubated with the microalgae lysate. The data regarding the adsorption of GFP for three BION-to-GFP mass ratios (1:1, 3:1 and 6:1) for different initial protein concentrations in solution are represented in Figure 3a. The different protein concentrations should indicate if, for the same mass ratio, the total concentration in the system might have an impact on the final protein capacity on the nanoparticles.

Here again, as in Figure 1a and as expected, increasing the BION-to-GFP mass ratios leads to increased protein adsorption from the solution. While, at the lowest BION-to-GFP mass ratio (1:1), only 13% GFP is adsorbed for a GFP concentration in solution of 1 g L^−1^, 49% adsorption is observed for 3:1 and complete protein adsorption is observed for 6:1. The data in Figure 3a also highlight that lower initial protein concentrations result in higher adsorbed protein percentage for the mass ratios 1:1 and 3:1, while for 6:1 almost all proteins are adsorbed for the tested protein concentrations in solution. For the lowest BION-to-GFP mass ratio, the adsorbed protein percentage depends strongly on the total concentration in the system. This might have to do with agglomeration effects. One hypothesis is a connection between increasing protein concentration in the solution and higher tendency for proteins to agglomerate in the solution, reducing their total capacity on the solid adsorbent. However, pH shifts and analytical/experimental limitations (e.g., because of sample dilution for better quantification) might also offer an explanation.

After washing the BIONs-GFP, the solids were incubated with microalgal lysate in ASW media for 30 min., and then the protein concentrations in the solution were quantified (Figure 3b). Compared to pure GFP adsorption directly onto the bare BIONs, here we see a slightly reduced adsorption capacity of the solids at BION-to-protein mass ratios 6:1 and 3:1. For the 1:1 BION-to-protein mass ratio, a maximum additional protein adsorption of only 10–20% is observed for the different starting protein concentrations in the incubated suspension. Higher solid concentrations lead to substantially higher adsorbed protein percentages; however, no total protein separation is possible. In contrast to the previous situation with GFP alone (Figure 3a), changes in the protein concentrations in solution did not markedly impact here the percentage of separated proteins for a specific BION concentration. The data demonstrate that the presence of a previous GFP corona does not impede the further adsorption of proteins onto the bio–nano surface, but that the influence of the nanoparticle-to-protein mass ratio, i.e., the available surface for adsorption compared to the number of possible adsorbate molecules, is decisive for a larger uptake.

The microalgae lysate contains a high number of different biomolecules that also interact with the BIONs as part of the corona [23]. Some of them might have occupied a portion of the available adsorption sites on the surface [11,12,14,15,23], or might have played a role in stabilizing a fraction of the proteins in the solution, reducing the overall ability of proteins to adsorb onto the surface. Nevertheless, observing the “intermediate” BION-to-protein mass ratio of 3:1, the presence of other molecules should also promote greater protein adsorption than pure protein as long as enough surface is accessible for both proteins and other molecules. The lack of enough available surface seems to be the most suitable explanation for the reduced capacity of the 1:1 BION-to-protein mass ratio.

Using spectroscopic fluorescence measurements of the supernatants after incubation of the BION-GFP corona with the lysate, the desorption of GFP from the nanoparticles in these experiments could be measured (see green dots in Figure 3b). Based on the GFP desorption percentages, an increase in the BION-to-protein mass ratio abruptly decreases desorption of GFP from the corona. This fact confirms that, at a 1:1 BION-to-protein mass ratio, there is not sufficient surface area for all adsorbate molecules. For the initial protein concentration in the lysate of 1 g L^−1^, up to 90% of the GFP desorbs for the ratio 1:1, while only 19% and 2% desorption are observed for the ratios 3:1 and 6:1, respectively. However, the degree of GFP desorption is very low when the initial lysate protein concentration is only 0.25 g L^−1^ for all three BION-to-protein mass ratios. This lower desorption could be related to a higher degree of agglomeration of the solids at higher protein concentrations. It may also simply confirm that there is an additional effect from the increasing protein concentration in the system with a protein agglomeration mechanism in the solution.

For the lowest GFP values, fluorescence was found to be under the reliable detection limit, and thus the data can only be interpreted as a tendency. Additionally, the emission spectra of GFP and chlorophyll overlap at 515 nm. Accordingly, desorption could be measured only by blanking with a representative empty value of the standard solutions of the lysate at 515 nm. However, with SDS-PAGE of the final supernatants, we visualized the tendency of the data (see Figure S4 and Table S1 for quantification of band intensities; dilution factors must be considered). The PAGE corroborates the behavior pattern observed from the quantification of proteins and suggests further protein uptake and GFP desorption from the soft corona taking place in the presence of new proteins in the solution. Importantly, even if we did not quantify the two washing steps, we nevertheless observed no drastic color or turbidity change in the supernatants after the washing. Therefore, we presume that no strong protein loss took place during washing.

Fluorescence microscopy is an elegant tool for observing the effects discussed above. The images in Figure 3c and Figures S6–S9 show the final corona after the sequential adsorption, i.e., the BION-GFP-lysate corona for the incubation of 0.75 g L^−1^ BIONs, first with 0.25 g L^−1^ GFP and, after washing, with 0.25 g L^−1^ lysate protein suspension. In Figure 3c, the overlap of the particle spots with the fluorescence of the GFP and the red fluorescence of the chlorophyll is observable and confirms the presence of both molecules, GFP and chlorophyll, on the BION. For enhanced quality and for completeness, Figures S5–S8 in the Supplementary Materials show the brightfield image (Figure S5), the green fluorescence of GFP on the BION-GFP-lysate corona (Figure S6), the red fluorescence of the chlorophyll on the same sample (Figure S7) and Figure 3c again (Figure S8), enlarged. The images corroborate that both GFP and lysate components accumulate onto the BIONs.

**Figure 4:**
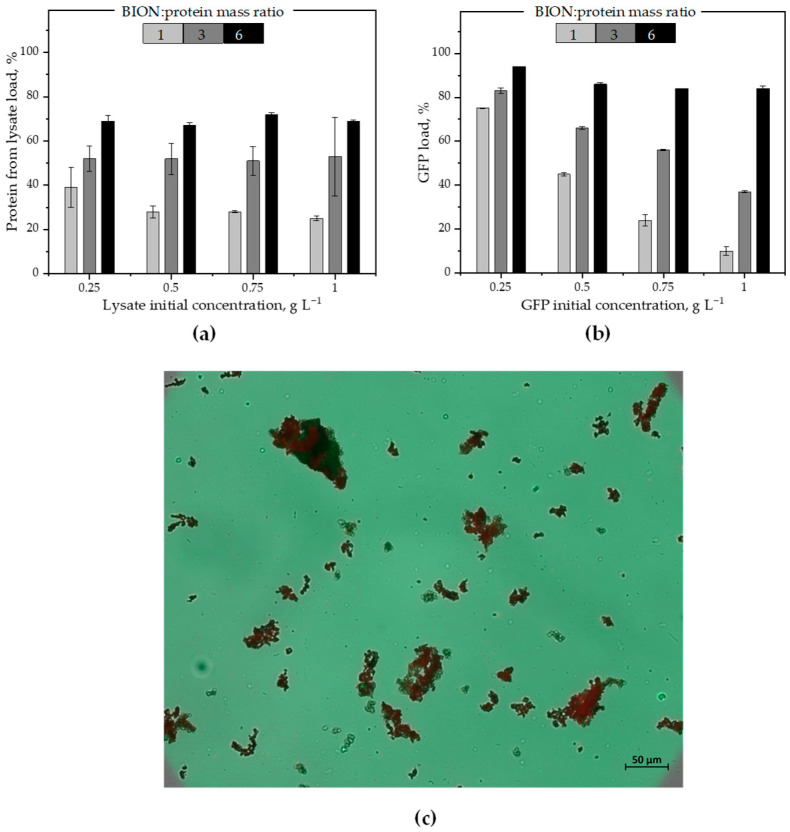
(**a**) Percentage of total protein adsorption from microalgal lysate onto BIONs for different initial total protein concentrations in suspension and for three different BION-to-lysate protein mass ratios. The adsorption value are related to the initial protein masses in each system. (**b**) Percentage of GFP adsorption after incubation of the BIONs-lysate with GFP for the three BION-to-lysate protein mass ratios from (**a**). (**c**) Microscopy images of BIONs (6 g L^−1^) first incubated with microalgae lysate (1 g L^−1^) and then with GFP (1 g L^−1^) with 200× magnification. Overlap of brightfield (to observe the position of the BION agglomerates) with 508 nm fluorescence (GFP, in green) and with 685 nm fluorescence (chlorophyll, in red). Scale bar 50 µm.

The next set of experiments was designed to examine the opposite approach: the incubation first of BIONs with the microalgae lysate and, afterward, the incubation of the BION-lysate corona in the GFP suspension. Here, we first coated the nanoparticles with biomolecules from the lysate, then washed the solids twice and subsequently incubated them in the GFP suspension. The data regarding the first step, the adsorption of the protein from a microalgae lysate to three BION-to-lysate protein mass ratios (1:1, 3:1 and 6:1), is represented graphically in Figure 4a. The data show a slight decrease in the percentage of protein adsorption for the 1:1 mass ratio, while the lysate protein concentration in the solution increases, similarly to the GFP adsorption case, but less abruptly, possibly due to the lack of available surface for the higher quantity of proteins in the solution. For the mass ratios 3:1 and 6:1, the percentage of separated protein is constant for the lysate protein concentrations and is higher the more binding places are available in the suspension, i.e., higher for the 6:1 mass ratio than for the 3:1 ratio. The more binding places, the greater the percentage of separated proteins, but, in contrast to the GFP situation, no total protein separation was possible here. This probably happened because other molecules also occupy part of the binding places, even if some of these other molecules might help stabilize the proteins in solution and therefore reduce their tendency to partition onto the solid adsorbent. These results again demonstrate that, in complex mixtures, the degree of protein adsorption depends primarily on the adsorbent-to-protein mass: the more particles, the higher the percentage of adsorbed proteins.

Little research exists on complex mixtures; most of the available information relates to individual molecule adsorption onto a variety of nanoparticles [14,73,74,75,76]. From previous work on individual molecule adsorption in a model mixture of biomolecules [59], we learned how strongly other molecule types impact the adsorption of a protein onto a surface. Some articles from the last few years quantify total protein adsorption from complex mixtures, mainly from blood plasma or serum. Yet, they seldom compare the impact of the surface-to-target concentration ratio. We hypothesize from the knowledge gained in previous work with a model mixture [23] that agglomeration in a solution might impact the protein capacity on the nanoparticles. However, the main effect we observe in our data is the impact of the saturation of binding places, and therefore the general lower percentage of adsorbed proteins for higher protein quantities in solution and lower nanoparticle concentrations. One important question that remains open in our minds is how the observed effects affect the picture of a corona divided into a soft (multilayer) and a hard corona (first monolayer on the solid surface). It seems more plausible to imagine only one corona in some places of agglomerated biomass, but in general of only one monolayer, as the images in Figure 1 suggest.

In the subsequent step, after two washing steps, we incubated the BIONs with the adsorbed lysate corona (BIONs-lysate) at different GFP concentrations for 30 min. Our data in Figure 4b again confirm that higher BION-to-protein mass ratios and lower initial GFP concentrations in the system increase the percentage of total GFP adsorbed. For the 1:1 mass ratio, adsorption rises from 10% to approx. 75% as the initial protein concentration decreases. For the highest BION-to-protein mass ratio (6:1), a very high uptake of GFP onto the corona takes place for all initial protein concentrations in solution, potentially due to the presence of sufficient surface for adsorption. These results show that, after establishing an equilibrium, the protein corona for both types of experiments (the one-protein corona and the complex corona) is still dynamic and can adsorb additional proteins (and desorb others) from the biological suspension subsequently added until a new equilibrium is established. The higher the BION-to-protein mass ratio, the higher the total protein adsorption. Based on the data, we expect that, for any given protein concentration, there is a corresponding BION concentration where almost total protein recovery should be possible.

We used here again fluorescence microscopy to validate the corona composition. The image in Figure 4c shows the final corona after the sequential adsorption, i.e., the final BION-lysate-GFP corona for the incubation of 6 g L^−1^ BIONs with microalgae lysate concentration of 1 g L^−1^ and, after two washing steps, the subsequent incubation with GFP at 1 g L^−1^. Figure 4c is the result of overlapping the brightfield with both fluorescence images, and substantiates that both GFP and lysate components form the BION corona. For completeness, the same image in a larger size can be found in the Supplementary Materials (Figure S9). Both microscopy series, i.e., in Figure 3 and Figure 4, show similar results, regardless of the sequence of incubation. The agglomerates vary in size, with some far surpassing 50 µm. All the agglomerates show the characteristic fluorescence of both chlorophyll from the *M. salina* and GFP, indicating that both coronae are dynamic and still adsorb further biomolecules and release others when incubated in a new media. The high salinity of the microalgae environment probably facilitates the inhomogeneous formation of agglomerates of different sizes and the strong molecule intake.

Regarding all sequential data (Figure 3 and Figure 4), the amount of GFP adsorption appears to be quite similar with or without preliminary incubation with lysate for the mass ratios 1 and 3. For the 6:1 BION-to-protein mass ratio, while the total GFP was separated onto the bare BIONs, a slightly lower quantity was separated onto the BION-lysate corona. Regarding the lysate proteins, the GFP-corona seems to lead to an increase in the protein adsorption for the mass ratios 3:1 and 6:1 series compared to the direct adsorption onto the nanoparticles. In contrast, a decrease is observable for the 1:1 mass ratio, probably because the surface is already fully occupied by GFP. The role that agglomeration effects play in these experiments should be clarified in the future.

To validate the data from the sequential experiments, we carried out SDS-PAGEs of the final supernatants at the end of the sequential incubation experiments (see Figure S10). The gels corroborate qualitatively the tendencies of the BCA and fluorescence measurements in Figure 3a,b and Figure 4a,b. There is a clear and significant reduction in the band intensity in all the supernatants, both for GFP and lysate proteins, as the BION-to-protein mass ratio increases, i.e., for large BION concentrations and low protein concentrations in solution.

## 3. Materials and Methods

As we describe at the end of the introduction, this manuscript includes data to answer three questions, all of them related to the impact of the nanoparticle-to-protein masses on the quantity of proteins separated:

- In Section 2.1, two iron oxides with different oxidation degrees and clearly different colors, black magnetite and brown maghemite, are used to compare their ability to separate proteins.
- In Section 2.2, the separated protein bands from the magnetite sample from Section 2.1 are compared to the protein bands remaining in solution through analysis of SDS-gels.
- Section 2.3 builds the core of the manuscript and presents data upon sequential adsorption of GFP and of lysate proteins onto the BIONs (i.e., the magnetite nanoparticles). This last section reinforces the impact of the nanoparticle-to-protein ratios on the nanoparticles’ adsorption capacity for proteins for different total molecule concentrations in the system and for a low nanoparticle-to-protein ratio (below the monolayer saturation value) compared to higher ratios.

### 3.1. Nanoparticle Synthesis and Characterization

Details on particle synthesis, as well as the characterization methods, are summarized in the Supplementary Materials. Before incubation with biological material, the particles were stored in artificial seawater, ASW (see Supplementary Materials for composition details).

### 3.2. Green Fluorescent Protein

Details on the production and purification of the Green Fluorescent Protein (GFP) can be found elsewhere [65]. Briefly, the protein was overexpressed in *E. coli* BL21 (DE3) at 37 °C in Riesenberg medium in a fed-batch process. For the downstream processing, plate separation, high-pressure homogenization and depth filtration were applied. To concentrate and purify the (His)_6_-GFP up to >95%, cross-flow filtration, anion-exchange chromatography and affinity chromatography were carried out. The GFP obtained was stored at −20 °C in imidazole. Before use, the protein was rebuffered and transferred to ASW medium: 15 mL of the GFP solution was centrifuged in Vivaspin^®^ Turbo 15 ultrafiltration units (Sartorius AG, Göttingen, Germany) at 4000× *g* and 4 °C for 45 min. The liquid phase was discarded, and centrifugation was repeated three times with 15 mL ASW medium. The filter cake was resuspended in 15 mL ASW medium, and the concentration of GFP was measured via bicinchoninic acid (BCA) assay and spectroscopic fluorescence measurements (see below).

### 3.3. Microchloropsis Salina

The *M. salina*, microalgae cell biomass (SAG 40.85) was provided by the Chair of Biochemical Engineering of the Technical University of Munich. The original strain came from the Culture Collection of Algae at the University of Göttingen, Germany. The microalgae were cultivated in open thin-layer cascade photobioreactors and harvested in a growth-limiting ASW medium as described elsewhere [33,35,77,78]. The algal suspension was collected during the stationary phase, and was placed on the windowsill in Schott jars with the lid half open at room temperature and in laboratory light. The ASW medium was not changed. The algae were subject to daily fluctuations as a living system.

Measurement of the biomass concentration was carried out gravimetrically from the filter cake after filtration and washing of the algae (to eliminate residual salt) for every batch of microalgae. The concentration of the algal suspension was obtained from the Chair of Biochemical Engineering of the Technical University of Munich and confirmed in our chair spectrophotometrically (Biospectrophotometer, Eppendorf AG, Hamburg, Germany) in technical triplicates and based on standard curves of the optical density at 750 nm. For some experiments, the microalgae were concentrated in advance: 50 mL of the microalgae suspension was centrifuged at 3200× *g* (Heraeus Megafuge 16-R centrifuge, Thermo Fisher Scientific GmbH, Schwerte, Germany) for 10 min. at room temperature. The supernatant was removed, and the pellet resuspended with 10 mL ASW medium. Subsequently, the concentration was measured again spectrophotometrically. Further details on the microalgae production and separation can be found in the Supplementary Materials.

### 3.4. Microalgal Cell Lysis

For the *M. salina* disruption, glass beads (0.25–0.5 mm, Carl Roth GmbH + Co. KG, Karlsruhe, Germany) were loaded into the 1 mL mark in 2 mL reaction vials, and 1 mL microalgae suspension was then added. The supernatant was disrupted in the mixer mill MM400 (Retsch GmbH, Haan, Germany) for 15 min. at a frequency of 25 s^−1^. TEM images of the lysate suspension can be found in the Supplementary Materials Figure S2. Subsequently, the protein concentration was determined using a BCA assay.

### 3.5. Batch Adsorption of Microalgal Lysate onto Nanoparticles

Cells were disrupted shortly before the incubation with nanoparticles. The lysates (500 µL) were then mixed with the corresponding nanoparticle suspension in the necessary volumes depending on the target nanoparticle-to-protein or nanoparticle-to-biomass ratios and incubated for 10 min. at 25 °C and 1000 rpm in the thermomixer. The particles were separated from the supernatant using a neodymium hand magnet (0.5 T). The protein concentration in the supernatants was measured via a BCA assay. The protein load on the BIONs was determined by subtracting the supernatant values from the original lysate values. The solids were then washed twice with distilled water, and adsorbed protein was also directly quantified from the solids via a BCA assay of the solid phase. The experiments were conducted in technical duplicates (two independent experiments) with analytical triplicates (three replicates quantified using UV-Vis spectroscopy) of each independent sample unless otherwise specified. Error bars represent the standard deviation of the six values. For the experiments corresponding to Figure 1, one incubation took place for each point, and three analytical replicates of each incubation were measured via UV-Vis.

### 3.6. Batch Adsorption of GFP onto BIONs

BION suspensions were prepared from dilutions of the stock solution and mixed with the dilutions of the re-buffered GFP in ASW medium. Therefore, 1 mL of each of these dilutions was incubated with 1 mL of the different BION concentrations on the Thermomixer comfort (Eppendorf AG, Hamburg, Germany) for 10 min. at 1000 rpm and 25 °C. The BIONs were separated magnetically, the supernatant was removed, and the concentration of the supernatant was measured via fluorescence (see below). The experiments were carried out in technical duplicates, and analytical triplicates of each individual sample were measured.

### 3.7. Sequential Batch Adsorption Experiments

Sequential experiments were conducted in two ways. In the first series, GFP was initially incubated with the BIONs, followed by the addition of microalgal lysate to the solids from the first incubation step. In the second series, microalgal lysate was first incubated with the BIONs, and, subsequently, the solids were incubated with GFP. Both series were performed using analogous procedures. For both series, nanoparticle-to-biomass ratios were based on the total protein concentration in the biomass samples rather than the algae biomass concentration to maintain consistency with the ratios used for the pure GFP samples.

The chosen concentrations of GFP as well as the total protein concentrations of the microalgal lysate were 1, 0.75, 0.50 and 0.25 g L^−1^. Each concentration of the GFP was incubated with BIONs in final mass ratios 1:1, 3:1 and 6:1 BION-to-GFP, respectively. In the first step, 500 µL of the protein solution (GFP or microalgal lysate, depending on the experimental series) were incubated with 500 µL of the BION suspension in a 2 mL reaction vessel for 10 min. at 25 °C and 1000 rpm in a thermomixer. The BIONs were separated using a hand magnet. The supernatants were transferred to a new reaction vessel and stored at 4 °C for further analysis. The solids were then washed twice with 1 mL ASW medium, and the wash solution was discarded. An amount of 1 mL of the second protein solution, with concentrations of 1, 0.75, 0.50 and 0.25 g L^−1^, was added to each BION ratio. The BIONs were detached from the reaction vessel wall by brief vortexing in the Vortex Gene2 vortexer (Scientific Industries Inc., Bohemia, Czech Republic) before incubating the reaction vessels in a thermomixer for 30 min. at 25 °C and 1000 rpm. For subsequent microscopic analysis, 30 µL of the solution was siphoned off and transferred to a new reaction tube. The rest of the BIONs were separated employing a hand magnet, and the supernatant was removed and transferred to a new reaction vessel for later analysis.

GFP concentrations were determined spectroscopically by fluorescence measurement at an excitation wavelength of 485 nm; emissions were measured at 515 nm. The protein concentrations of the pure lysate and the lysate supernatants after incubation with GFP, as well as the initial solutions of the diluted lysates, were determined via BCA assay. The supernatants of the lysates after incubation with GFP, and the GFP supernatants after incubation with the microalgal lysate, were also analyzed via SDS-PAGE.

Each incubation was carried out in technical duplicates, and analytical triplicates of each individual incubation were measured.

### 3.8. Total Protein Quantification by BCA Assay

Bovine serum albumin (BSA) was employed as standard protein for the BCA assays. Calibration curves were prepared from 2 g L^−1^ BSA stock solutions in ASW medium. A Nunc 96-well microplate (ref. 260836, Thermo Fisher Scientific) was pipetted with 25 µL of the different samples (standard solutions, ASW medium for the blank in duplicates, or the individual samples in triplicates), incubated at 37 °C for 30 min. and shaken in the Multiwell Reader Infinite^®^ 200 PRO Series (Tecan Deutschland GmbH, Crailsheim, Germany) for 30 s. Absorbance was measured with UV-VIS spectroscopy at 562 nm, and then the total protein concentration was calculated based on the calibration line. See Supplementary Materials for further details.

### 3.9. GFP Quantification via Fluorescence Measurement

After rebuffering GFP to ASW medium, calibration curves in the range between 0.025 and 2 g L^−1^ were prepared. Samples were measured in black 96-well plates (Brand plates -F-, pureGrade, ref. 781608, Brand GmbH + CO KG, Wertheim, Germany) after shaking for 15 s in a multiwell reader. The plates were loaded with 100 µL of the separated supernatants in triplicate, 100 µL of the standard dilutions and 100 µL of ASW medium as a blank in duplicates. The excitation wavelength was 485 nm. The emission was measured at 515 nm. The concentration of the supernatants was determined based on the calibration line.

### 3.10. Protein Visualization via SDS-PAGE

SDS-PAGE (Sodium Dodecyl Sulfate Polyacrylamide Gel Electrophoresis) was used to control the purity of protein bands and for validation purposes. The procedure was conducted via two successive gels, a 15% resolving gel and a 5% stacking gel. To better identify the sample pockets, the stacking gel was stained with bromophenol blue (Carl Roth GmbH + Co KG, Germany). Samples were prepared by mixing loading buffer with 10% 1 M 1,4-dithiothreitol (DTT). Subsequently, the samples were denaturated for 5 min. at 95 °C in a thermomixer. Next, 4 µL of a Color Prestained Protein Standard (NEB GmbH, Frankfurt a. Main, Germany) and 10 µL of the samples were added to the pockets of the gel. Electrophoretic separation was carried out using the SDS-PAGE system Mini Gel Tank (Thermo Fisher Scientific GmbH, Schwerte, Germany) at 160 V for 70 min. (Voltage Source: Major Science, Saratoga, CA, USA). The gels were then stained for 30 min. in a staining solution containing Coomassie Brilliant Blue R 250 (Carl Roth GmbH + Co. KG, Karlsruhe, Germany) and then decolorized in a solution consisting of 10% (*v/v*) ethanol and 30% (*v/v*) acetic acid. After overnight storage, the gels were transferred to dd-water with 500 µL of staining solution. Staining, decolorizing and rinsing steps were carried out with a Mini-Shaker Multi Bio 3 D shaker (Biosan, Riga, Latvia). The gels were scanned with the Amersham Typhoon NIR Plus (GE Healthcare Europe GmbH, Freiburg, Germany).

A slightly modified method was followed for the selectivity demonstration in Figure 1e: 12% polyacrylamide was employed as the resolving gel and 5% as the stacking gel. To facilitate band comparison, 5 or 10 kDa Eppendorf centrifugal concentrators were used to adjust the protein concentrations of the adsorption supernatants to that of the non-contacted lysate. Particle concentration was also modified by adjusting the volume in which they were resuspended after the experiments: 10 µL of either the particles or the supernatants were mixed with 10 µL of loading buffer supplemented with DTT and heated to 95 °C for 5 min. before loading. The gels were scanned using an Amersham Typhoon NIR Plus (GE Healthcare Europe GmbH, Freiburg, Germany).

### 3.11. Optical Characterization of Solid Phases by Light Microscopy

For microscopy images, 20 µL samples were deposited on object slides and overlaid with a cover slip. Subsequently, the supernatant was observed in the Axio Observer inverted microscope (Carl Zeiss Microscopy GmbH, Jena, Germany) with the 20 mm objective. For every selected area, one brightfield image and two fluorescence images were taken. The images were taken at a wavelength of 508 nm to detect GFP, and at a wavelength of 685 nm to detect chlorophyll. Using the microscope software, we first viewed the images individually and then in combined constellations: “Brightfield + GFP”, “Brightfield + chlorophyll” and a third combination consisting of all three modes overlapped.

### 3.12. Transmission Electron Microscopy Imaging

Transmission electron microscopy (TEM) was used to visualize the nanoparticles in saline media, the lysate and the corona after incubation of both the lysate and the nanoparticles. In all cases, 10 µL samples with a concentration of approx. 0.01 g L^−1^ were deposited onto a glow-discharged carbon-coated copper grid (Science Services, Berlin, Germany) and let dry. No samples were stained. Images were recorded with a Tecnai G2 Spirit (Thermo Fischer Scientific/FEI, Eindhoven, The Netherlands) TEM device and processed using ImageJ software v1.53e.

For further details on the experimental methods see the Supplementary Materials.

## 4. Conclusions

Back in 2014, Del Pino et al. had argued for the importance of quantitative data on corona formation [79]. Recently, Han and Domaille emphasized the value of predefining a protein corona as a strategic approach for employing nanoparticles in biomedicine [80]. However, the general literature still rarely addresses the critical role of the experimental conditions and, in particular, the concentration ratio of adsorbent-to-adsorbate, i.e., the ratio of surface area available to the number of binding molecules. In this work, we present data on the separation ability of nanoparticles pre-coated with a one-protein or a complex corona to separate proteins. Moreover, our findings support previous research highlighting the significance of the adsorbent-to-adsorbate ratio on both the total amount of protein that can be separated and the overall evolution of the bio–nano corona [23,81]. Additionally, the data demonstrate that the level of surface oxidation for superparamagnetic iron oxide does not significantly influence the evolution of the separated protein mass, at least in the context of microalgae lysate in artificial seawater media. Proteins can be completely separated from a suspension by adjusting the nanoparticle concentration, regardless of whether the particles are oxidized. Sequential adsorption experiments on a one-protein or on a complex corona show only a slight reduction in the capacity for protein separation, indicating that corona formation does not hinder further protein adsorption.

Furthermore, this study confirms that even unmodified, non-functionalized materials such as bare BIONs show a kind of selectivity for some proteins from a biotechnological lysate, provided the adsorbent-to-adsorbate ratio is sufficiently low. This selectivity might be due to differences in the direct interaction with the surface, in the agglomeration behavior, in interactions with the corona layer(s) or with molecules in the solution. Despite their potential, studies on biotechnological coronae remain scarce. Most nanoparticle corona studies focus either on simplified model systems using one or a few proteins in buffered environments [82], or on physiological coronae formed in animal in vitro or in vivo systems [83] Recently, the scope of corona research has expanded to include biological units, such as small extracellular vesicles [84], enriching the field. Increased investigation into coronae formed from complex lysates derived from bacteria, fungi, algae and other microorganisms would significantly advance our understanding of adsorption processes in bioseparation and related areas. Moreover, as Vianello et al. noted, harnessing coronae for industrial use depends on understanding surface coordination chemistry and its underlying molecular mechanisms [85].

Systematic methodologies are essential for working with complex, natural samples. Here, more experimental guidance would benefit the study of adsorption in real-world environments. In this context, nanoparticles offer promising tools for enhancing selectivity and expediting advanced analytical application [86]. Robust analytical methods to determine biomolecule distributions across different phases and mixtures are essential for the efficient exploitation of biomass in future biorefinery strategies. As demonstrated in this study, nanoparticles can serve as effective carrier materials in such approaches, provided that safety and ecological impacts are carefully considered [87,88].

We believe that corona formation holds vast potential for future technological applications. The concept of a “personalized nanoparticle-protein corona” [89] may also become a compelling topic in bioseparation. Future research should investigate the potential of magnetic particles coated with natural biopolymers as a possible means to enhance biocompatibility and cellular internalization and reduce agglomeration issues [90,91].

## Data Availability

The original contributions presented in this study are included in the article/Supplementary Materials. Further inquiries can be directed to the corresponding author.

## Supplementary Materials

The following supporting information can be downloaded at https://www.mdpi.com/article/10.3390/ijms26188995/s1. Refs. [33,35,45,48,77,78] are cited in the supplementary materials.
