# Supplementary material for: Bio Meets Nano: Protein Exchange in Saline Biocoronae on Magnetic Nanoparticles
Source: Int J Mol Sci. 2025 Sep 16;26(18):8995. doi: 10.3390/ijms26188995 (PMC12469985; doi:10.3390/ijms26188995)
Supplement: Supplementary file 1 [file ijms-26-08995-s001.zip › ijms-3821386-supplementary.pdf]

International Journal of
Molecular Sciences

MDPI

Supporting Materials

Bio Meets Nano: Protein Exchange in Saline Biocorona on Magnetic Nanoparticles

Paula Fraga-García [1,*], Sandra Haßelt [1], Carlos Eduardo Díaz-Cano [1], Lucía Abarca-Cabrera [1], Yasmin Kaveh-Baghbaderani [1], Sebastian P. Schwaminger [2,3], Massimo Kube [4], Hendrik Dietz [4]

[1] Chair of Bioseparation Engineering, Department of Energy & Process Engineering, School of Engineering & Design, Technical University of Munich, 85748 Garching, Germany; sandra.hasselt@gmail.com (S.H.); cadiazcano@gmail.com (C.E.D.-C.); l.abarca@tum.de (L.A.-C.); y.kaveh@tum.de (Y.K.-B.)
[2] NanoLab, Division of Medicinal Chemistry, Otto-Loewi Research Center, Medical University of Graz, 8010 Graz, Austria; sebastian.schwaminger@medunigraz.at
[3] BioTechMed-Graz, 8010 Graz, Austria
[4] Lehrstuhl für Biomolekulare Nanotechnologie, Department of Biosciences, School of Natural Sciences, Technical University of Munich, 885748 Garching, Germany; massimo.kube@tum.de (M.K.); dietz@tum.de (H.D.)
* Correspondence: p.fraga@tum.de; Tel.: +498928915759

Materials

Magnetite nanoparticles (BIONs) synthesis and characterization

Here we present the most relevant characteristics of the BIONs (bare iron oxide nanoparticles; this is the abbreviation we established some years ago for the black, non-oxidized and non-coated iron oxide nanoparticles we work with). The BIONs were synthesized by co-precipitation of Fe^{2+} and Fe^{3+} aqueous salt solutions in an alkaline environment as described by Roth *et al.* [1]. Therefore, 200 mL of ferrous chloride (100 mmol; $FeCl_2{\cdot}4H_2O$ from Bernd Kraft GmbH, Germany), 200 mL of ferric chloride (200 mmol; $FeCl_3{\cdot}6H_2O$ from AppliChem GmbH, Germany) and 500 mL sodium hydroxide (1 mol; NaOH from AppliChem GmbH, Germany) were prepared with degassed and deionised water. The co-precipitation of magnetite nanoparticles was performed in a stirred tank reactor under nitrogen atmosphere to prevent oxidation of the precursors and the product. After synthesis, the suspension was washed several times with degassed and deionized water to lower the conductivity. The particle suspension was then stored in water: 72 g L^{-1}, pH 7.8, conductivity <179 µS/cm.

The particles were then characterized as seen in Figure S1. The particle size was determined through measurements of X Ray Diffraction (XRD) and Transmission Electron Microscopy (TEM). For XRD, freeze-dried samples were investigated with a Stadi-P diffractometer with a molybdenum Kα source (λ=0.7093 Å) from STOE & Cie GmbH (Germany) at room temperature in transmission geometry. For an evaluation of the particle size the Scherrer equation was used:

$$d = \frac{K \cdot \lambda}{\Delta 2\theta \cdot \cos\theta}$$

Here the diameter d can be calculated from the broadening of reflections $\Delta 2\theta$ and the angle of the reflection $\cos\theta$ (the 311 reflection has been used). K is a form factor for spherical particles (0.89), λ is the wavelength of the source. The data was fitted with the gaussian model and particle size value was calculated as an average of the 5 main peaks of the particles in the XRD. This resulted in a value of 9.2 nm for the BIONs. TEM pictures were produced with a JEOL JEM-1400 Plus transmission electron microscope (JEOL, Germany). The suspensions were dropcasted on a carbon coated copper grid. For each sample

three images were evaluated and 30 particles were counted per picture for the statistical investigation. Using the program Image J, 35 particles in each of three TEM images were measured. The values obtained were used to calculate the size distribution presented in Figure S1. The value of mean particle size was determined by fitting to a Gaussian distribution in the program Origin Pro and was determined to be 8.9 nm. The XRD diffraction pattern points out to a superparamagnetic iron oxide. For the determination of magnetic properties the SQUID magnetometer MPMS XL-7 by Quantum Design (San Diego) was used. The freeze-dried samples were measured in changing fields from +50 kOe to -50 kOe at 300 K and rendered a value of ~ 70 $Am^2\ kg^{-1}$, Furthermore, the specific surface area of the BIONs was determined by nitrogen adsorption using a Micrometrics Gemini VII (Micromeritics GmbH, Germany). The resulting value of 78 $m^2\ g^{-1}$ enables us to know the available surface area of the BIONs for interaction with the media. Further, the particles were characterized by Fourier-transform infrared spectroscopy (FTIR) in an ALPHA II (Bruker Optics, Germany) FTIR spectrometer and the matching Platinum attenuated total reflection (ATR) module. Sixty-four scans per sample and measurement were performed. The baseline was subtracted via the rubber band method in the software OPUS.

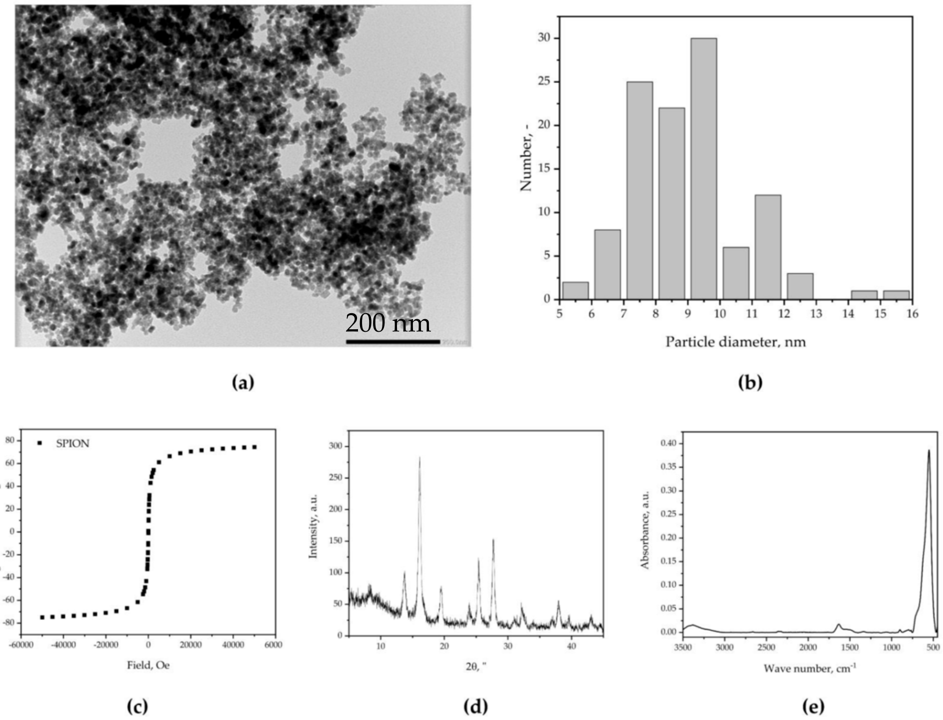

Figure S1. BION Characterization. (a) TEM image of the magnetite nanoparticles, (b) histogram of particle sizes from 3 different TEM images with 30 particles counted out of each image, (c) hysteresis curve of magnetite from -50 kOe to +50 kOe at 300 K, (d) X-ray diffractogram, and (e) ATR-FTIR spectrum of BIONs.

Maghemite nanoparticles synthesis and characterization

The maghemite material was a strongly oxidized magnetite sample. Magnetite was synthesised as described by Roth et al. [1] by the co-precipitation of Fe^{2+} and Fe^{3+} aqueous salt solutions in an alkaline environment, exactly in the same way as described in the section above. The magnetite suspension was washed several times with degassed and deionised water to remove extraneous ions until the conductivity dropped below 200 μS/cm. Afterwards, a mild oxidation of magnetite was performed in a double-walled glass reactor as described before [2]. Briefly: 1 L of a suspension of freshly prepared magnetite nanoparticles with a concentration of 7 g L^{-1} was stirred at 400 rpm and heated to 60 °C. After having maintained the temperature constant for 10 min., compressed air was bubbled through the reaction mixture. Nitric acid (HNO_3) purchased from Merck KGaA, Germany,

was utilised as oxidising agent for the harsh oxidation conditions. Therefore, 700 mL of the magnetite (7 g) nitric acid (490 mmol) suspension was stirred at 250 rpm and held at 60 °C for 24 hours.

All details on the particles′ characterization have been published elsewhere before [2]. Main properties of the particles are: (a) a median particle diameter of 9.4 and 7.3 nm from TEM and XRD analysis, respectively and a saturation magnetization of approx. 66 emu g^{-1}. The specific surface area was 93 m^2 g^{-1}. The clear oxidation of the material was confirmed by Raman, X-ray photoelectron and Mössbauer spectroscopy.

Microalgae and Artificial Sea Water (ASW)

The experiments were carried out with *Microchloropsis salina* (SAG 40.85). The microalgae species were cultivated and provided by the Institute of Biochemical Engineering of the Technical University of Munich. Cultivation took place in open thin-layer cascade photobioreactors in Artificial Sea Water (ASW) medium (27 g L^{-1} NaCl, 6.6 g L^{-1} $MgSO_4$ * 7 H_2O, 1.5 g L^{-1} $CaCl_2$ * 2 H_2O, 1.0 g L^{-1} KNO_3, 0.07 g L^{-1} KH_2PO_4, 0.021 g L^{-1} Na_2EDTA * 2 H_2O, 0.014 g L^{-1} $FeCl_3$ * 6 H_2O). All chemical agents used were of analytical grade or higher. The interaction studies were performed with microalgal cells in the stationary growth phase, after 12-18 days of cultivation or longer. The microalgal concentration after cultivation was between 5 and 40 g L^{-1}. Details on the cultivation can be found in works from Apel *et al*. [3] and Schädler *et al*. [4–6].

Prior to the experiments, changes in the cultivation media were evaluated through pH, conductivity and optical density measurement of the biological suspensions. In general, the conductivity was close to 45 ± 2 mS cm^{-1} and the pH slightly alkaline. The absorbance of the initial sample before incubation with the BIONs was also measured (OD_0). Extrapolation of absorbance values to cell densities was carried out based on a calibration curve for the measuring range of algae concentrations. Light microscope pictures of the algae were conducted in an Axio Observer 7 (Carl Zeiss AG, Oberkochen, Germany) microscope.

For several experiments the microalgae needed to be concentrated to achieve the necessary protein concentration. Therefore, 50 mL of the microalgal suspension was centrifuged at 3200 g for 10 min. at room temperature (25 °C) using the Heraeus Megafuge 16-R centrifuge (Thermo Fisher Scientific GmbH, Schwerte, Germany). The supernatant was removed and the pellet resuspended with 10 mL of ASW medium. Subsequently, the concentration was determined.

The lysate was obtained from cell milling using glass beads. The beads were filled to the 1 mL mark in 2 mL reaction tubes. Subsequently, 1 mL of microalgae suspension was added. Digestion of the microalgae was carried out in the MM400 vibrating mill (Retsch GmbH, Haan, Germany) for 15 min. at a frequency of 25 s^{-1}. The supernatant was then lifted off the glass beads and transferred to a new reaction tube. Figure S2 shows images of the cell lysate after lysis and bead separation. The supernatants were pooled and the protein concentration determined by bicinchoninic acid (BCA) assay.

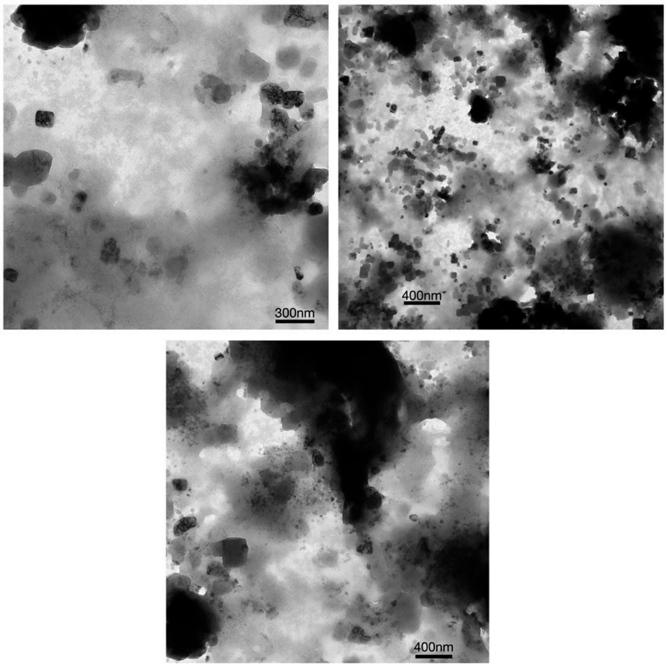

Figure S2. Image of the cell debris after cell milling and separation from the milling beads.

Experimental procedures

The biomass represents in all experiments the remaining mass after filtration of the microalgae twice with a high water volume. This was done to get the cell mass free of salt ions: washing leads to the separation of the salts from the cultivation media.

To investigate the adsorption capacity of GFP onto the BIONs, dilutions were prepared from the rebuffered GFP using ASW medium; 1 mL of each of these dilutions was incubated with 1 mL of the different BIONs concentrations on Thermomixer comfort (Eppendorf AG, Hamburg, Germany) for 10 min. at 1000 rpm and 25 °C. The BIONs were separated using a Neodym hand magnet (0.5 T); the supernatant was lifted off, and the concentration of the supernatant was determined via fluorescence measurement.

The fluorescence of GFP results from the spatial arrangement of the three amino acids Ser65, Tyr66 and Gly67. A chromophore forms in the protein, which fluoresces when excited with blue or UV light. The excitation wavelength of GFP is 395 nm and 475 nm, respectively, and emission occurs at 508 nm. Standard dilutions of the protein were prepared from the re-buffered stock solution using ASW medium. The detection limit for GFP in this assay ranged from 0.025 to 2.000 g L^{-1}. For measurement of the samples, 100 μL of the separated supernatants in triplicate were added to the 96 well plate; 100 μL of the standard dilutions and 100 μL of ASW medium as blank in duplicate were added to the 96 well plate. The plate was shaken for 15 sec in the multiwell reader. The excitation wavelength was chosen at 485 nm. The emission was measured at 515 nm. The concentration of the supernatants was calculated using the previously prepared calibration line.

Total protein concentration determination was carried using a bicinchoninic acid (BCA) assay, which is a colorimetric based on the reduction of Cu^{2+} to Cu^{+}; Cu^{+} reacts then with bicinchoninic acid to form a purple color complex whose absorbance can be measured at 562 nm. Bovine serum albumin (BSA) was used as the reference protein. For the quantification, a calibration curve ranging from 0.025 to 2.000 g L^{-1} was prepared from a 2 g L^{-1} stock solution. In a 96 well plate, 25 μL of the standard solutions and 25 μL of ASW

medium were pipetted for blanks in duplicate and 25 µL for samples in triplicates. The working reagent was prepared from commercial reagent A and B in a 50 to 1 ratio immediately before use. The required reagents were taken from the PierceTM BCA Protein Assay Kit (Thermo Fisher Scientific GmbH, Germany); 200 µL of working reagent was added to the standard, blank, and samples. To minimize the error due to the intrinsic color of chlorophyll, an additional 25 µL of the sample was prepared with 200 µL of ASW medium in triplicate, the value of which was later subtracted from the sample value. For these samples, a blank value was also included with 225 µL of ASW medium in duplicate determination. The 96 well plate was incubated at 37 °C for 30 min. The plate was shaken in the Multiwell Reader Infinite® 200 PRO Series (Tecan Deutschland GmbH, Germany) for 30 s and adsorption was subsequently measured at 562 nm. The total protein concentration was calculated using the calibration line.

SDS-PAGE (Sodium Dodecyl Sulfate Polyacrylamide Gel Electrophoresis) was used for purity control, to detect proteins or protein fragments and to make rough estimations of protein molecular weights. Proteins were separated using two successive polyacrylamide gels. In the stacking gel, protein fractions migrate more quickly and are concentrated into a narrow band, facilitating sharper separation. The actual separation of proteins occurs more slowly in the subsequent resolving gel. The polyacrylamide gels, consisting of a 15% resolving gel and a 5% stacking gel, were cast prior to use. Polymerization was initiated by adding 10% ammonium persulfate (APS) and TEMED. The stacking gel was stained with bromophenol blue to better identify the sample pockets. The sample was prepared by mixing with a 2-fold loading buffer (4% SDS, 20% (v/v) glycerol, 4.8% (w/v) bromophenol blue in 150 mM Tris buffer pH 6.8) mixed in a 1:1 ratio and the addition of 10% 1 M 1,4-dithiothreitol (DTT). The exact composition of the 15% resolving gel was 30% acrylamide, 1.5 M Tris pH 8.8, 10% SDS, 10% APS TEMED and ddH2O, while for the 5% stacking gel it was 30% acrylamide, 1 M Tris pH 6.8, 10% SDS, 10% APS, TEMED and dd H2O. Subsequently, the samples were denatured for 5 min at 95 °C in a thermomixer. Denaturation with the anionic surfactant SDS leads to a negative charge of the proteins and ensures their mobility in the gel. The addition of DTT leads to a reduction of disulfide bridges so that the proteins are linear

Results

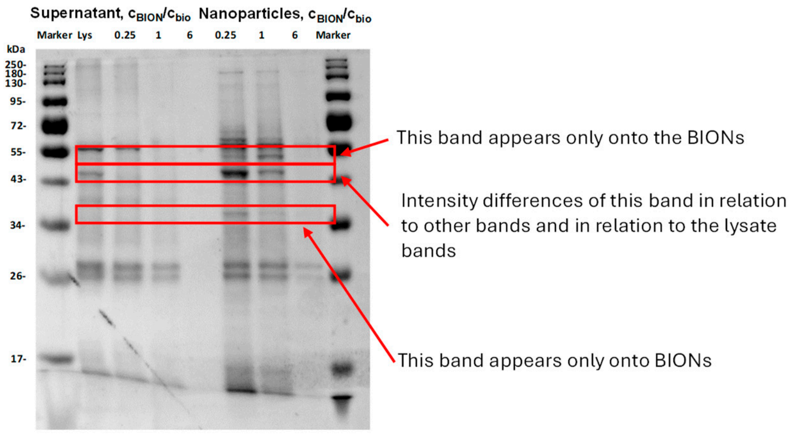

Figure S3. Copy of Figure 1(e) where some bands have been marked to make clearer the selectivity issue. There are significant differences in the preferential adsorption of several protein bands.

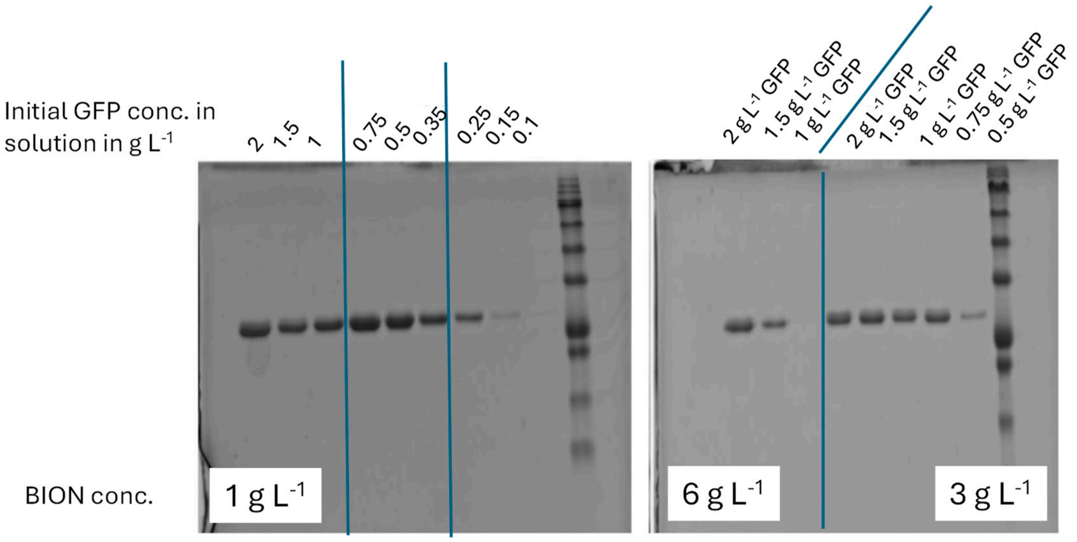

Figure S4. SDS gels of the protein contents in the supernatants after GFP adsorption onto the BIONs. Only the supernatants where protein was still detectable through fluorescence measurements are shown. On the left side, supernatants of the incubations with 1 g L^{-1} BIONs. Starting on well 2, from left to right, the concentrations decrease from 2 g L^{-1} to 0.1 g L^{-1} as initial concentration. (b) Supernatants of the incubation with 6 g L^{-1} BIONs (wells 3 to 5 with proteins from 2 g L^{-1} to 1 g L^{-1} initial concentration in solution) and with 3 g L^{-1} BIONs (wells 6 to 10 with protein from 2 g L^{-1} to 0.5 g L^{-1}). For the supernatants of the incubations with 1 and 3 g L^{-1} BIONs respectively, the samples between 2 g L^{-1} and 1 g L^{-1} initial GFP concentration were diluted to avoid overloading the wells. Lane 4 is empty due to the low concentration of the sample in the supernatant. The number of nanoparticles is so high compared to the protein quantity in the system that all GFP molecules are adsorbed at this initial concentration. Any GFP that might remain in solution is below the lowest intensity recognizable in the SDS gel bands. The corresponding data in Figure 3 (a) further corroborate that the GFP is completely adsorbed at 6:1 nanoparticle-to-protein mass ratios for 1 g/L initial protein concentration.

Table S1. Quantification of the intensity of the GFP bands. For the supernatants of the incubations with 1 and 3 g L^{-1} BIONs respectively, the samples between 2 g L^{-1} and 1 g L^{-1} initial GFP concentration were diluted to avoid overloading the wells. To confirm the concentration values in solution, 2-fold multiplication of these values (marked in red) is needed. Additionally, for correlation with the isotherm values, the solids´ concentration should be considered.

**1 g L^{-1} BIONs**		**3 g L^{-1} BIONs**		**6 g L^{-1} BIONs**	
GFP conc. (g L^{-1})	Band intensity	GFP conc. (g L^{-1})	Band intensity	GFP conc. (g L^{-1})	Band intensity
2	79	2	57	2	70
1.50	59	1.5	62	1.5	35
1	68	1	49	1	N/A
0.75	99		56		
0.5	85		16		
0.35	58		N/A		
0.25	35				
0.15	8				
0.1	N/A				

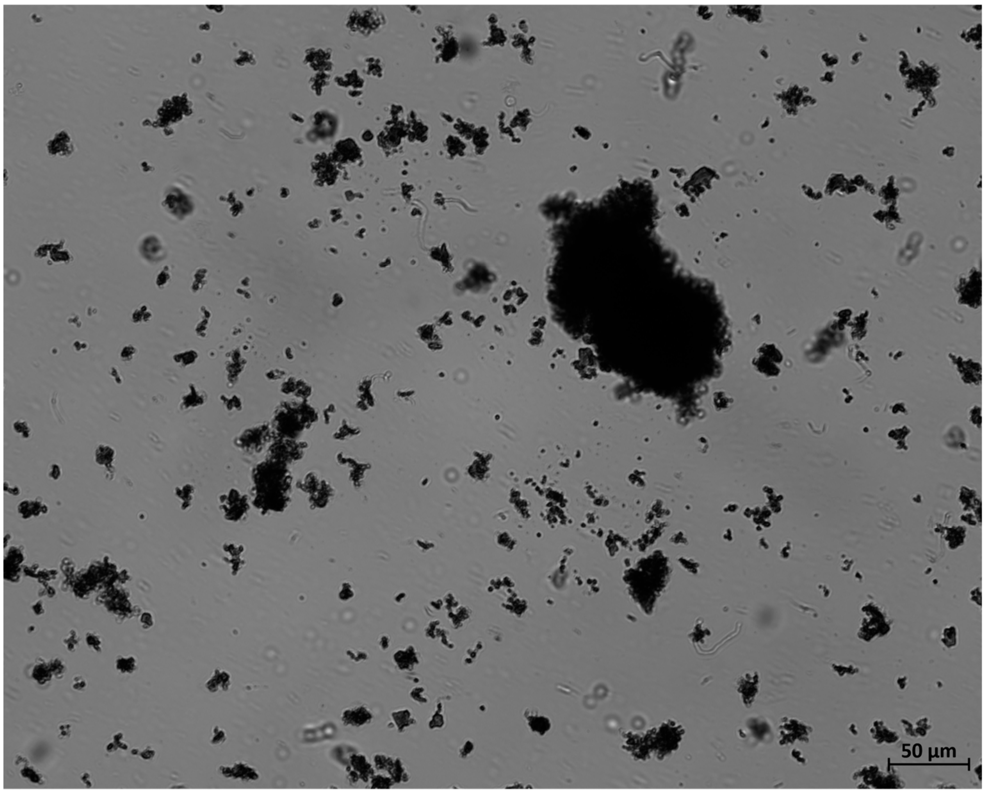

Figure S5. Microscopy images of the final solid sample for the BION-to-GFP mass ratio 3:1 first incubated with GFP (0.25 $g_{protein}$ L^{-1}) and then with microalgae lysate (0.25 $g_{protein}$ L^{-1}) at 200x magnification. O). Brightfield image to see the particle agglomerates.

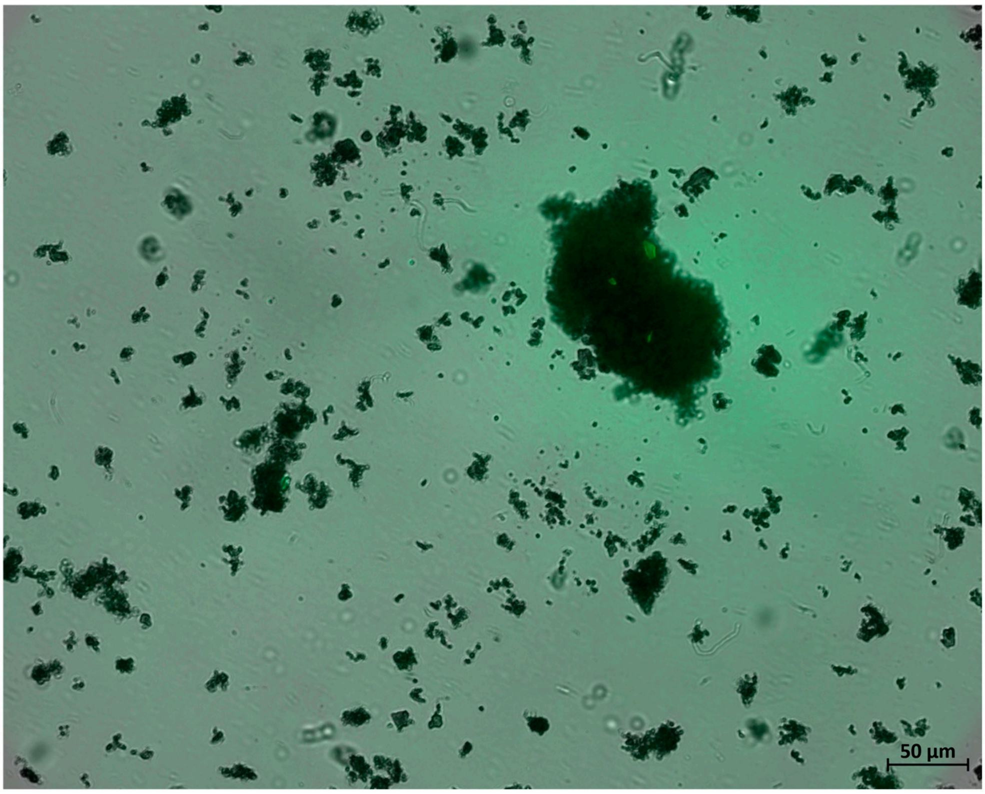

Figure S6. Microscopy image of the final solid sample for the BION-to-GFP mass ratio 3:1 first incubated with GFP (0.25 $g_{protein}$ L^{-1}) and then with microalgae lysate (0.25 $g_{protein}$ L^{-1}) at 200x magnification. Image in brightfield (to see the particle agglomerates) overlapped with 508 nm fluorescence (to see GFP, in green).

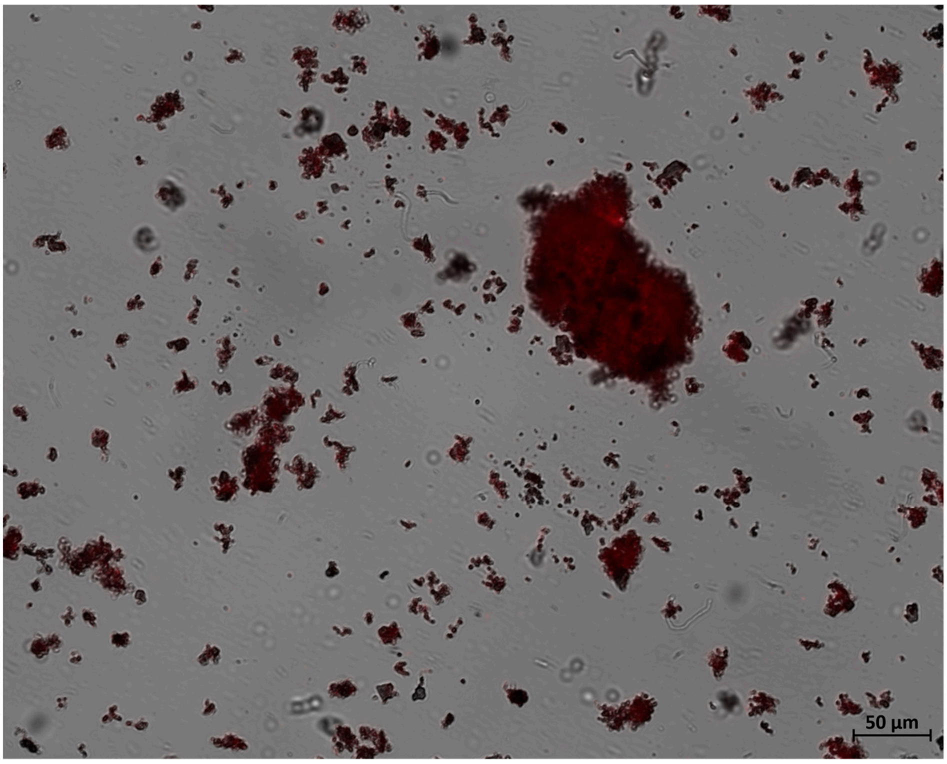

Figure S7. Microscopy image of the final solid sample for the BION-to-GFP mass ratio 3:1 first incubated with GFP (0.25 $g_{protein}$ L^{-1}) and then with microalgae lysate (0.25 $g_{protein}$ L^{-1}) at 200x magnification. Image in brightfield (to see the particle agglomerates) overlapped with 685 nm fluorescence (to see Chlorophyll, in red).

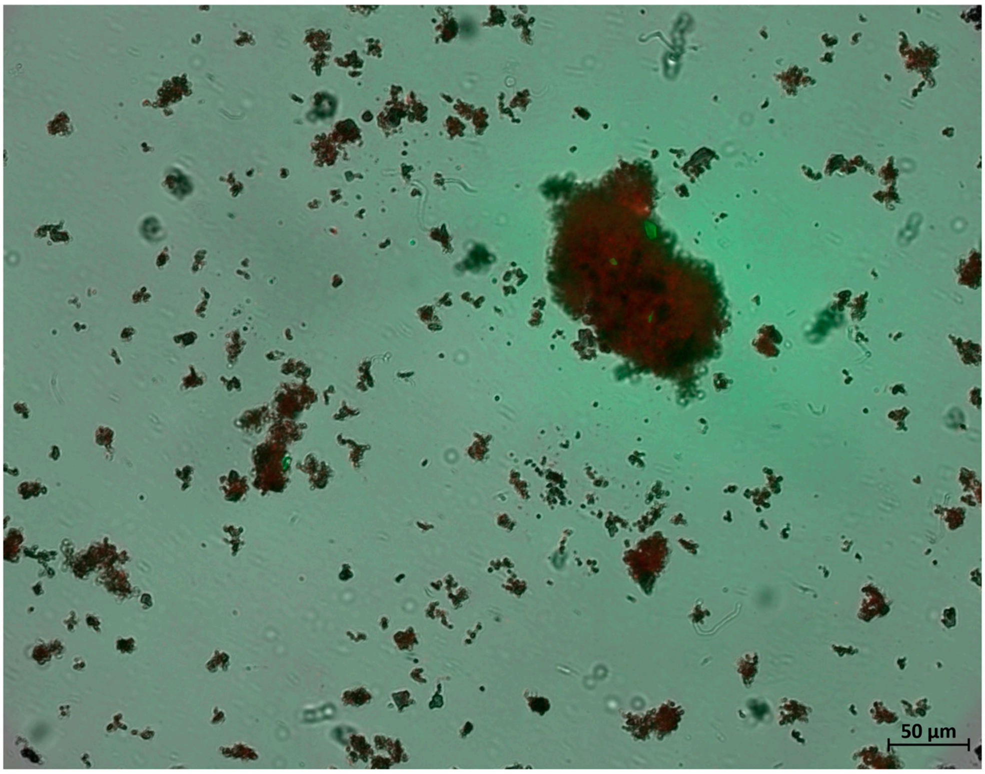

Figure S8. Microscopy image of the final solid sample for the BION-to-GFP mass ratio 3:1 first incubated with GFP (0.25 $g_{protein}$ L^{-1}) and then with microalgae lysate (0.25 $g_{protein}$ L^{-1}) at 200x magnification. Image in brightfield (to see the particle agglomerates) overlapped with 508 nm fluorescence (to see GFP, in green) and with 685 nm fluorescence (to see Chlorophyll, in red).

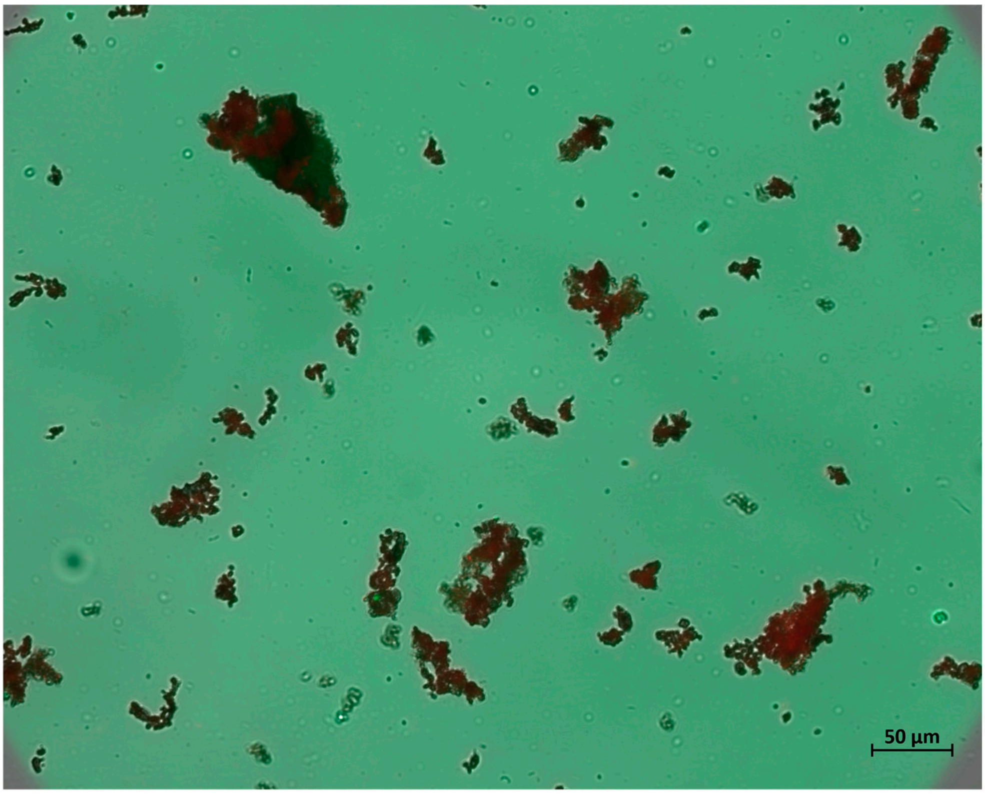

Figure S9. Microscopy image of BIONs (6 g L^{-1}) first incubated with microalgae lysate (1 g L^{-1}) and then with GFP (1 g L^{-1}) with 200x magnification. Image in brightfield (to see the particle agglomerates) overlapped with 508 nm fluorescence (to see GFP, in green) and with 685 nm fluorescence (to see Chlorophyll, in red).

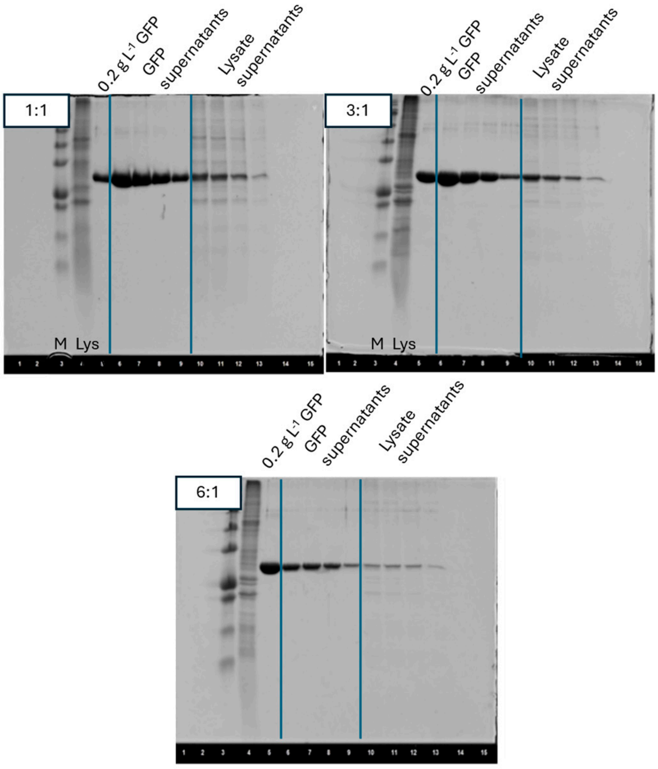

Figure S10. SDS-PAGEs of the final supernatants from the sequential incubation experiments. Every gel corresponds to the BION-to-protein initial mass ratio indicated in the upper left side of each gel

(1, 3 and 6 g g^{-1} respectively). Each gel includes a protein standard (lane 3), the original algae lysate (lane 4) without any dilution, the GFP solution in a concentration of 0.2 g L^{-1} (lane 5), the GFP supernatants after incubating GFP with BION-lysate coronae (decreasing protein concentrations 1, 0.75, 0.5, 0.25 g L^{-1} in wells 6-9) and the lysate supernatants after incubating microalgal lysate with BION-GFP coronae (decreasing protein concentrations 1, 0.75, 0.5, 0.25 g L^{-1} in wells 10-13). Pockets 1, 2 and 14 remained empty.

The lysate supernatants after incubating microalgal lysate with GFP-BION coronae (Figure S4, lanes 10 to 13, from highest total concentration in well 10 to lowest in well 13) substantiate the desorption of GFP from the corona (after washing). Here comparing the lanes´ intensities with the intensity of the GFP reference in lane 4 (0.2 g L^{-1}) is very helpful. Lanes 6 to 9 correspond to the GFP supernatants after incubating GFP with the lysate-BION coronae (decreasing protein concentrations from well 6 to well 9). These lanes suggest that there was very little desorption from the lysate-BION corona, although GFP is adsorbed. The very thin bands seen for larger proteins seem to indicate some contamination in the original GFP suspension (they are also observable in the GFP reference lane).

Datasets to the figures with examples of calibration lines

Core data to Figure 1(a).

	Maghemite		Magnetite	
**MNP-to-biomass mass ratio**	Adsorption [%]	Std dev	Adsorption [%]	Std dev
0.5	24.0	1.9	18.4	2.1
1	34.0	0.9	36.7	1.2
2.5	72.2	1.1	65.2	0.9
6	100	0	99.3	0.3

BCA calibration line for maghemite data to Figure 1(a), as a BCA example.

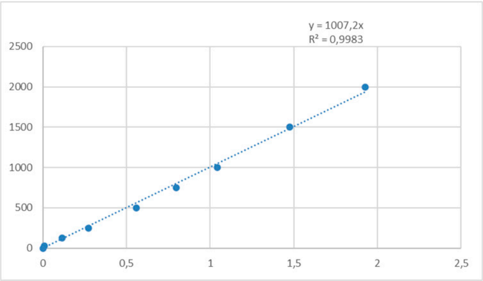

Data to Figure 2(a). Values correspond to the mass of protein adsorbed on the BIONs in percentage.

	BION-to-biomass mass ratio				
	**1**	**2.5**	**3**	**5**	**6**
**Biomass [g/L]**					
**1.5**	47	68	72	80	81
**1.8**	40	67	69	81	82
**8.2**	44	85	90	94	94
**9.6**	33	73	79	89	94

Data to Figure 2(b) and representative fluorescence calibration line for the 1 g/L BION dataset.

**1 g/L BION**											
C_{eq} [mg/L]	1937	1246	942	533	270	125	74	11	9	0	0
Ads prot [mg/g]	63	254	58	217	230	225	176	139	91	50	25
Std dev [mg/g]	4.9	49.9	3.5	4.9	0.7	2.3	1.5	1.2	3.7	0.1	0.1
**3 g/L BION**											
C_{eq} [mg/L]	1300	1042	427	169	37	10	1	0	0	0	0
Ads prot [mg/g]	233	153	191	194	154	113	83	50	33	17	8
Std dev [mg/g]	18.4	45.1	0.8	6.1	6.9	0.5	0.1	0.3	0.2	0	0
**6 g/L BION**											
C_{eq} [mg/L]	564	173	10	1	0	0	0	3	0	0	0
Ads prot [mg/g]	238	219	165	125	83	58	42	25	17	8	4
Std dev [mg/g]	5.7	9.9	0.9	0.2	0.1	0.1	0.1	1.6	0.2	0.2	0.1

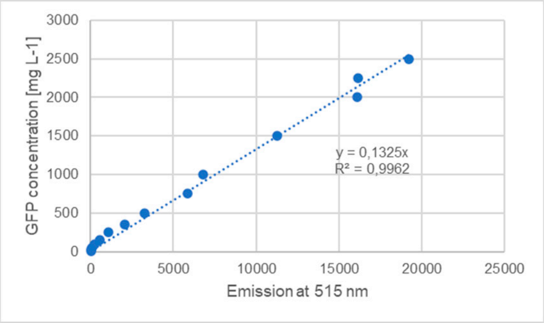

Data to Figures 3(a) and 3(b).

	GFP				Lysate			
**BION-to-protein mass ratio 1**	1 g/L	0.75 g/L	0.5 g/L	0.25 g/L	1 g/L	0.75 g/L	0.5 g/L	0.25 g/L
Protein Ads [%]	13	24	40	67	19	8	4	14
Std dev [%]	1	0	0.2	1.3	0.3	4.8	0	0.9
**BION-to-protein mass ratio 3**	1 g/L	0.75 g/L	0.5 g/L	0.25 g/L	1 g/L	0.75 g/L	0.5 g/L	0.25 g/L
Protein Ads [%]	49	55	62	77	67	75	76	72
Std dev [%]	0.4	0.5	0.9	1.1	2.2	0.8	0.8	1.8
**BION-to-protein mass ratio 6**	1 g/L	0.75 g/L	0.5 g/L	0.25 g/L	1 g/L	0.75 g/L	0.5 g/L	0.25 g/L
Protein Ads [%]	100	99	98	86	81	86	86	83
Std dev [%]	0	0.4	0	0.1	1.9	0.9	0.4	0.4

	GFP				Lysate			
**BION-to-protein mass ratio 1**	1 g/L	0.75 g/L	0.5 g/L	0.25 g/L	1 g/L	0.75 g/L	0.5 g/L	0.25 g/L
Protein Ads [%]	13	24	40	67	19	8	4	14
Std dev [%]	2	0,0	0.2	1.3	0.3	4.8	0	0.9
**BION-to-protein mass ratio 3**	1 g/L	0.75 g/L	0.5 g/L	0.25 g/L	1 g/L	0.75 g/L	0.5 g/L	0.25 g/L
Protein Ads [%]	49	55	62	77	67	75	76	72
Std dev [%]	0.4	0.5	0.9	1.1	2.2	0.8	0.8	1.8
**BION-to-protein mass ratio 6**	1 g/L	0.75 g/L	0.5 g/L	0.25 g/L	1 g/L	0.75 g/L	0.5 g/L	0.25 g/L
Protein Ads [%]	100	99	98	96	81	86	86	83
Std dev [%]	0	0.4	0	0.1	1.9	0.9	0.4	0.4

Data to Figures 4(a) and 4(b).

	Lysate				GFP			
**BION-to-protein mass ratio 1**	1 g/L	0.75 g/L	0.5 g/L	0.25 g/L	1 g/L	0.75 g/L	0.5 g/L	0.25 g/L
Protein Ads [%]	25	28	28	39	10	24	45	75
Std dev [%]	1	0.5	2.7	9.0	1.9	2.7	0.8	0.1
**BION-to-protein mass ratio 3**	1 g/L	0.75 g/L	0.5 g/L	0.25 g/L	1 g/L	0.75 g/L	0.5 g/L	0.25 g/L
Protein Ads [%]	53	51	52	52	37	56	66	83
Std dev [%]	17.8	6.4	7.1	5.7	0.4	0.3	0.6	1.3
**BION-to-protein mass ratio 6**	1 g/L	0.75 g/L	0.5 g/L	0.25 g/L	1 g/L	0.75 g/L	0.5 g/L	0.25 g/L
Protein Ads [%]	69	72	67	69	84	84	86	94
Std dev [%]	0.6	0.9	1.3	2.5	1.2	0.1	0.7	0.1

Representative calibration line for the BCA assay quantification (left side) and for the GFP fluorescence quantification (right side) corresponding to the data in Figures 3 and 4.

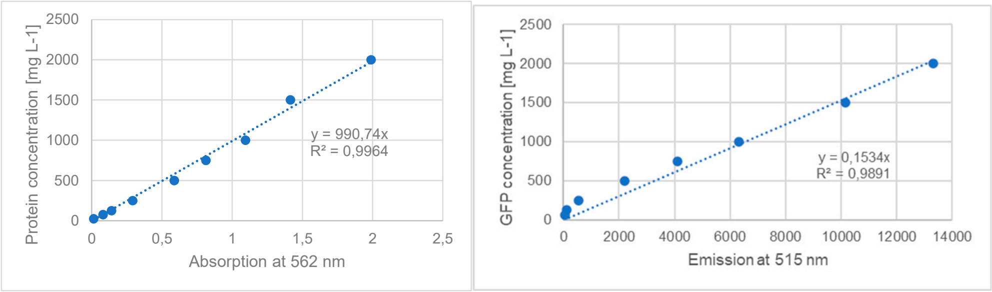

References

1. Roth, H.-C.; Schwaminger, S.P.; Schindler, M.; Wagner, F.E.; Berensmeier, S. Influencing factors in the CO-precipitation process of superparamagnetic iron oxide nano particles: A model based study. *Journal of Magnetism and Magnetic Materials* **2015**, *377*, 81–89, doi:10.1016/j.jmmm.2014.10.074.
2. Schwaminger, S.P.; Bauer, D.; Fraga-García, P.; Wagner, F.E.; Berensmeier, S. Oxidation of magnetite nanoparticles: impact on surface and crystal properties. *CrystEngComm* **2017**, *19*, 246–255, doi:10.1039/C6CE02421A.
3. Apel, A.C.; Pfaffinger, C.E.; Basedahl, N.; Mittwollen, N.; Göbel, J.; Sauter, J.; Brück, T.; Weuster-Botz, D. Open thin-layer cascade reactors for saline microalgae production evaluated in a physically simulated Mediterranean summer climate. *Algal Research* **2017**, *25*, 381–390, doi:10.1016/j.algal.2017.06.004.
4. Schädler, T.; Thurn, A.-L.; Brück, T.; Weuster-Botz, D. Continuous Production of Lipids with *Microchloropsis salina* in Open Thin-Layer Cascade Photobioreactors on a Pilot Scale. *Energies* **2021**, *14*, 500, doi:10.3390/en14020500.
5. Schädler, T.; Neumann-Cip, A.-C.; Wieland, K.; Glöckler, D.; Haisch, C.; Brück, T.; Weuster-Botz, D. High-Density Microalgae Cultivation in Open Thin-Layer Cascade Photobioreactors with Water Recycling. *Applied Sciences* **2020**, *10*, 3883, doi:10.3390/app10113883.
6. Schädler, T.; Caballero Cerbon, D.; Oliveira, L. de; Garbe, D.; Brück, T.; Weuster-Botz, D. Production of lipids with *Microchloropsis salina* in open thin-layer cascade photobioreactors. *Bioresour. Technol.* **2019**, *289*, 121682, doi:10.1016/j.biortech.2019.121682.
